# Hovendulcisic acid A-D: four novel ceanothane-type triterpenoids from *Hovenia dulcis* stems with anticancer properties

**DOI:** 10.3389/fchem.2024.1383886

**Published:** 2024-05-14

**Authors:** Jianzhan Yang, Wanna Cai, Fan Wu, Shengmei Xu, Xiaoqi Zhang, Bo Liu, Fangfang Xu

**Affiliations:** ^1^ Guangdong Provincial Key Laboratory of Research on Emergency in TCM, the Second Clinical College of Guangzhou University of Chinese Medicine, Guangzhou, China; ^2^ Department of Pharmacy, The First Affiliated Hospital of Shantou University Medical College, Shantou, China; ^3^ Guangdong Provincial Engineering Research Center for Modernization of TCM, College of Pharmacy, Jinan University, Guangzhou, China

**Keywords:** *Hovenia dulcis* Thunb, ceanothane-type triterpenoids, hovendulcisic acid, antitumor activity, quantum chemical calculations

## Abstract

Sixteen ceanothane-type triterpenoids, including four new compounds—hovendulcisic acids A–D (**1**–**4**) —were purified from the stems of *Hovenia dulcis* Thunb. The structures of **1**–**4** were confirmed by comprehensive means including ECD and quantum chemical calculations. Putative biosynthetic pathways of **1**–**16** were proposed, and **3**, **5**, and **15** exhibited antitumor activity on A549 and MDA-MB-231 cells.

## Introduction

*Hovenia dulcis* Thunb (Rhamnaceae family) is a traditional Chinese medicine, and many parts of this herb, including seeds, fruit, leaves, and stem, have been used to relieve alcohol toxicity and protect the liver in ancient medical books (Wang, 1958; Su, 1985; Flora of China Editorial Committee, 1982; Chinese materia medica editorial board, 1999; Wu, 2000). Research on the *Hovenia* genus has revealed its anti-tumor efficacy (Ji, 2003; Ji, et al., 2003; Zhang, 2017), with triterpenoids and flavonoids as their main bioactive constituents (Yoshikawa et al., 1992; Yoshikawa et al., 1998a; Kang et al., 2017; Xu, et al., 2020a; Xu, et al., 2020b; Cai, et al., 2021).

Previous studies revealed that the triterpenoid saponins from *H*. *dulcis* inhibited Nrf2 expression (Cai, et al., 2021), indicated potential anti-tumor sensitization activity (Lin et al., 2020). This discovery piqued our interest about the triterpenoid constituents of the *Hovenia* genus. We found the stems of *Hovenia* genus to be rich in triterpenoids by LC-MS experimentation. The chemical constituents from the stem of *H*. *dulcis* were thus isolated and identified in this study. As a result, four new triterpenoids of hovendulcisic acid A-D (**1**–**4**) were isolated from 70% alcohol extract by multi-column chromatography and HPLC methods. The activity of **1**–**4** on Nrf2 were measured by luciferase reporter gene assay, and the cell viability of **1**–**4** on MDA-MB-231 and A549 cells were tested by CCK8 method. This research serves as a reference for further study on the pharmacological effects and drug active substance basis of *H. dulcis*.

## Materials and methods

### General experimental procedures

NMR data were obtained with a Bruker AMX-600 (Germany). HRESIMS was performed on a Thermo LTQ Orbitrap-Discovery (United States). Acid hydrolysis results were analyzed by Agilent GC 7890A/5975C (United States). UV was recorded on a Hitachi U-2910 (Japan). IR were acquired using a Perkin Elmer Spectrum 400 (United States). Preparative HPLC used an Agilent 1260 instrument equipped with a Cosmosil ODS C_18_ column (5 *μ*m, 10 mm × 250 mm). Optical rotations were measured on a Rodolph Autopol Ⅰ Polarimeter (United States); column chromatographies were performed with 80–100 and 200–300 mesh silica gel (Qingdao Haiyang) and Sephadex LH-20 (Pharmacia, United States). TLC was carried out on GF254 plates (Qingdao Haiyang). Human MDA-MB-231 cells were purchased from ATCC (VA, United States). D-(+)-glucose and tert-butylhydroquinone (tBHQ) were bought from Sigma Chemical (MO, United States).

### Plant materials

The stems of *H. dulcis* Thunb. were collected in December 2020 in Zhen-an City, Shanxi Province and identified by pharmacist Ganshu She in the Guangdong Provincial Hospital of Chinese Medicine. The samples were authenticated as voucher number (202012001) and stored at the TCM storehouse in the Second Clinical College of the Guangzhou University of Chinese Medicine.

### Extraction and Isolation

A total of 30.0 kg of air-dried and mechanically powdered stems of *H. dulcis* were extracted by reflux using ethanol-H_2_O solvent (70:30, *v*/*v*) thrice and then concentrated to obtain residue. The crude extract was then suspended with water and successively extracted by EtOAc and n-BuOH. The EtOAc fraction (382.9 g) was isolated on a silica gel column and eluted with CHCl_2_/MeOH solvent system to obtain seven fractions (E1 – E7). Fraction E1 (0.77 g) was isolated via ODS column [MeOH/H_2_O (40 : 60–100 : 0)], obtaining four subfractions (E1−1 − E1-4). Subfraction E1-2 was separated on a semi-prepared HPLC [aqueous acetonitrile (3.0 mL/min, 99 : 1, *v*/*v*)] and obtained **15** (2 mg, *t*_R_ = 18.9 min). Fraction E2 (62.37 g) was isolated on a silica gel (300–400 mesh) column [PE/EtOAc (1:0, 10:1, 9:1, 6:1, 5:1, 4:1, 3:1, 2:1, 1:1, and 0:1)] with 15 subfractions (E2−1 − E2-15). E2-2 was repeatedly dissolved and recrystallized in CHCl_2_ and MeOH to obtain compound **16** (35.1 mg). E2-4 (201.0 mg) was separated on a Sephadex LH-20 column (MeOH) and then separated on a ODS column [MeOH/H_2_O solvent (2:3–1:0)] to divide into four subfractions (E2-4–1 − E2-4–4). Compound **3** (19.1 mg, *t*_R_ = 12.1 min) was isolated from subfraction E2-4-2 by semi-prepared HPLC using aqueous acetonitrile (3.0 mL/min, 99 : 1, *v*/*v*). E2-6 (781.0 mg) was separated on a Sephadex LH-20 column (MeOH), and compound **5** (66 mg) was obtained by MeOH redissolution. E2-9 (1.11 g) fractions were separated by a silica gel column with CHCl_2_/MeOH (100:0, 250:1, 200:1, 150:1, 100:1, 50:1, 30:1, 10:1, 0:100). Compound **11** (38 mg, *t*_R_ = 20.5 min) was obtained from E2-9-6 by semi-prepared HPLC (acetonitrile: water, 65:35, *v*/*v*). E2-10 (2.60 g) was separated on a Sephadex LH-20 column (MeOH), obtaining five subfractions E2-10–1 − E2-10–5. E2-10–2 (268 mg) was isolated on an ODS column eluted with gradient MeOH/H_2_O solvent (20:80–100:0, *v*/*v*) to divide into six subfractions (E2-10-2–1 − E2-10-2–6). Compound **12** (2.2 mg, *t*_R_ = 9.0 min) was obtained from E2-10–2-2 by semi-prepared HPLC (acetonitrile: water, 67 : 33, *v*/*v*), and compound **8** (9.1 mg, *t*_R_ = 18.4 min) was obtained from E2-10–2-5 by semi-prepared HPLC (acetonitrile: water, 70 : 30, *v*/*v*). E2-12 (2.65 g) was separated on an ODS column [MeOH/H_2_O solvent (30:70–100:0, *v*/*v*)] to divide into six subfractions (E2-12–1 − E2-12–6). Compound **2** (5.1 mg, *t*_R_ = 10.6 min) was obtained from E2-12-4 by semi-prepared HPLC (acetonitrile: water, 75 : 25, *v*/*v*). Compound **7** (54 mg) was obtained from E2-12-5 by repeatedly dissolving and recrystallizing in MeOH. E2-13 (5.86 g) was separated on an ODS column [MeOH/H_2_O solvent (20 : 80–100 : 0, *v*/*v*)] to divide into eight subfractions (E2-13–1 − E2-13–8). Compound **9** (228.1 mg, *t*_R_ = 8.0 min) was obtained from E2-13-4 by semi-prepared HPLC (acetonitrile: water, 60 : 40, *v*/*v*). E2-13–6 was separated by silica gel column chromatography (CHCl_2_/MeOH, 100:0, 50:1, 40:1, 25:1, 15:1, 10:1, 5:1), obtaining five subfractions (E2-13-6–1 – E2-13-6–5). We obtained **10** (33.2 mg, *t*_R_ = 7.2 min) by semi-prepared HPLC (acetonitrile: water, 65:35, *v*/*v*) from E2-13-6–3. Fraction E3 (41.53 g) was separated on a silica gel (300–400 mesh) column eluted by CHCl_2_/MeOH (100:0, 25:1, 15:1, 10:1, 6:1, 4:1, 0:100) to obtain ten subfractions (E3−1 − E3-15). Subfraction E3-6 (2.73 g) was further separated on a Sephadex LH-20 column (MeOH) into eight subfractions (E3-6–1 − E3-6–8). Subfraction E3-6–2 (519.1 mg) was purified on an ODS column eluted with gradient MeOH/H_2_O (1:4–1:0), followed by semi-prepared HPLC eluted with acetonitrile/0.1% formic acid water (3.0 mL/min, 57 : 43, *v*/*v*) to obtain **4** (9.0 mg, *t*_R_ = 12.8 min). Compound **1** (5.1 mg) was isolated from subfraction E3-6–3 (591.1 mg) by an ODS column eluted with MeOH/H_2_O (1:4–1:0). Compound **13** (2.2 mg) was obtained from E3-6-5 by repeated dissolving and recrystallizing in MeOH. E3-8 (2.47 g) was separated on a Sephadex LH-20 column (MeOH) and semi-prepared HPLC (acetonitrile: water, 70 : 30, *v*/*v*), obtaining compound **14** (13.2 mg, *t*_R_ = 16 min).

### Spectroscopic data

Hovendulcisic acid A (**1**): white powder; m.p. 340–342°C; [*α*]^25^_D_ +11.20 (c 0.8, MeOH); UV (MeOH) *λ*_max_: 201.0 nm; IR (*ν*_max_ cm^−1^): 3330.0, 2955.3, 2868.3, 1457.3, 1392.8, 1241.0, 1715.6, 1682.6, 1644.6, 1174.0, and 883.5. HR-ESI-MS: *m/z* 533.3124 [M-H]^-^ (C_30_H_45_O_8_, calcd. for 533.3109). 1D NMR see Table 1 and Table 2.

**TABLE 1:** ^1^H NMR data of **1**–**4** (CD_3_OD, 600 MHz, *δ* in ppm, *J* in Hz).

Position	1^a^	2	3	4
	*δ*_H_	*δ*_H_	*δ*_H_	*δ*_H_
1	3.30, s	2.50, s	1.86, td, (8.2, 4.6)	2.48, s
2	-	-	4.47, dd, (11.5,4.5)	-
			4.04, dd, (11.5,7.8)	
3	4.87, s	4.08, s	4.94, d, (8.7)	4.07, s
5	2.22, m	1.56, m	1.22, m	1.58, m
6	1.50, m	1.43, m	1.46, m	1.39, m
	1.42, m	1.35, m	1.36, m	1.33, m
7	1.03, m	2.25, dt, (13.2, 3.3)	1.44, m	1.84, m
	1.94, m	1.06, m	1.42, m	1.81, m
9	2.80, d, (10.5)	1.61, m	1.69, dd, (12.9, 3.6)	1.52, m
11	2.38, m	1.78, m	1.57, m	1.64, m
	1.70, td, (9.0, 3.6)	1.67, m	1.53, m	1.77, m
12	3.20, m	1.82, m	1.75, m	1.55, m
	2.19, m	1.84, m	1.19, m	1.43, m
13	3.13, m	2.53, td, (12, 5.6)	2.30, td, (12.9,3.7)	2.52, td, (12.1, 5.3)
15	2.64, d, (12.4)	1.92, m	1.56, m	1.88, m
	2.01, m	1.42, m	1.16, m	1.49, m
16	2.16, m	1.59, m	2.25, dt, (12.8, 3.2)	2.35, dd, (10.1, 3.2)
	1.76, m	1.44, m	1.44, m	1.09, m
18	2.89, t, (8.6)	1.55, m	1.61, t, (11.4)	1.67, m
19	2.25, m	3.04, td, (10.8, 4.2)	3.02, td, (10.9, 4.7)	3.03, m
21	2.35, m	1.38, m	1.95, m	1.96, m
	1.99, m	1.94, m	1.40, m	1.37, m
22	2.99, d, (11.8)	1.36, m	1.90, m	1.36, m
	2.06, m	1.90, m	1.49, m	1.92, m
23	1.30, s	1.05, s	0.82, s	1.04, s
24	1.25, s	0.89, s	1.01, s	0.87, s
25	1.48, s	1.09, s	0.89, s	1.07, s
26	1.30, s	1.04, s	0.95, s	1.01, s
27	-	10.13, s	1.05, s	10.12, s
29	1.47, s	4.74, s	4.73, s	4.74, s
		4.63, s	4.61, s	4.62, s
30	1.39, s	1.70, s	1.71, s	1.69, s
1′			-	5.50, d, (8.0)
2′			2.05, s	3.31, m
3′			-	3.41, m
4′			1.98, s	3.36, m
5′				3.36, m
6′				3.82, m
				3.69, m

^a^ The solvent used was Pyridine-d5.

**TABLE 2:** ^13^C NMR data of **1**–**4** (CD_3_OD, 150 MHz, *δ* in ppm).

Position	1^a^	2	3	4
	*δ*_C_ type	*δ*_C_ type	*δ*_C_ type	*δ*_C_ type
1	67.6, CH	66.6, CH	57.6, CH	66.9, CH
2	178.4, C=O	178.2, C=O	66.6, CH_2_	178.6, C=O
3	84.9, CH	85.5, CH	88.1, CH	85.6, CH
4	44.1, C	44.2, C	40.8, C	44.2, C
5	57.4, CH	57.8, CH	63.1, CH	57.8, CH
6	19.5, CH_2_	19.5, CH_2_	19.0, CH_2_	19.5, CH_2_
7	38.8, CH_2_	33.7, CH_2_	35.5, CH_2_	26.2, CH_2_
8	42.4, C	44.1, C	43.1, C	44.2, C
9	46.5, CH	51.9, CH	51.8, CH	49.6, CH
10	50.6, C	50.4, C	46.0, C	50.3, C
11	25.4, CH_2_	24.9, CH_2_	24.8, CH_2_	24.9, CH_2_
12	30.9, CH_2_	26.2, CH_2_	26.4, CH_2_	35.4, CH_2_
13	41.4, CH	39.6, CH	39.3, CH	39.3, CH
14	60.1, C	59.0, C	43.9, C	58.9, C
15	29.7, CH_2_	25.5, CH_2_	31.0, CH_2_	25.4, CH_2_
16	38.1, CH_2_	35.6, CH_2_	33.4, CH_2_	33.1, CH_2_
17	61.3, C	56.9, C	57.3, C	57.4, C
18	50.4, CH	49.5, CH	50.5, CH	52.0, CH
19	52.7, CH	48.8, CH	48.6, CH	48.6, CH
20	72.9, C	151.4, C	151.9,C	151.3, C
21	29.6, CH_2_	31.5, CH_2_	31.7, CH_2_	37.4, CH_2_
22	35.6, CH_2_	38.1, CH_2_	38.2, CH_2_	31.3, CH_2_
23	31.7, CH_3_	31.2, CH_3_	25.6, CH_3_	31.3, CH_3_
24	20.6, CH_3_	19.7, CH_3_	24.9, CH_3_	19.7, CH_3_
25	20.1, CH_3_	19.4, CH_3_	14.9, CH_3_	19.6, CH_3_
26	19.2, CH_3_	17.9, CH_3_	17.4, CH_3_	17.9, CH_3_
27	179.2, C=O	211.3, C=O	15.1, CH_3_	211.4, CH
28	180.1, C=O	179.4, C=O	179.9,C=O	175.7, C=O
29	30.8, CH_3_	110.7, CH_2_	110.3, CH_2_	110.8, CH_2_
30	28.9, CH_3_	19.4, CH_3_	19.6, CH_3_	19.3, CH_3_
1′			172.8, C=O	95.2, CH
2′			21.0, CH_3_	74.1, CH
3′			172.7, C=O	78.4, CH
4′			20.9, CH_3_	71.1, CH
5′				78.9, CH
6′				62.3, CH_2_

^a^ The solvent used was Pyridine-d5.

Hovendulcisic acid B (**2**): white powder; m.p. 324–326°C; [*α*]^25^_D_ +16.0 (c 0.1, MeOH); UV (MeOH) *λ*_max_: 203.5 nm; IR (*ν*_max_ cm^−1^): 3475.3, 2968.4, 2937.8, 2868.3, 1454.1, 1402.4, 1379.8, 1722.0, 1718.9, 1687.9, 1644.6, 1235.4, and 883.5. HR-ESI-MS: *m/z* 499.3060 [M-H]^-^ (C_30_H_43_O_6_, calcd. for 499.3054). 1D NMR see Table 1 and Table 2.

Hovendulcisic acid C (**3**): white powder; m.p. 326–328°C; [*α*]^25^_D_ −11.8 (c 0.1, MeOH); UV (MeOH) *λ*_max_: 204.0 nm; IR (*ν*_max_ cm^−1^): 3397.0, 2944.8, 2869.0, 1739.6, 1701.3, 1458.2, 1375.0, 1235.1, 1186.2, 1029.0, and 774.7. HR-ESI-MS: *m/z* 555.3699 [M-H]^-^ (C_34_H_51_O_6_, calcd. for 555.3680). 1D NMR see Table 1 and Table 2.

Hovendulcisic acid D (**4**): white powder; m.p. 243.6–245.1°C; [*α*]^25^_D_ +15.12 (c 0.5, MeOH); UV (MeOH) *λ*_max_: 202.2 nm; IR (*ν*_max_ cm^−1^): 3391.4, 2947.8, 2868.3, 1748.0, 1722.0, 1698.9, 1641.4, 1065.5, 1028.0, and 892.3. HR-ESI-MS: *m/z* 661.3597 [M-H]^-^ (C_36_H_53_O_11_, calcd. for 661.3582). 1D NMR see Table 1 and Table 2.

### Acid hydrolysis and GC analysis

Acid hydrolysis and GC analysis of the glycoside were carried out according to Cai, et al. (2021). First, Compound **4** (2.1 mg) was hydrolyzed in HCl solution (2 M, 10 mL) in an oven (90°C, 4 h), and then evaporated to dryness. H_2_O was added to the residue and CHCl_3_ was used for extraction twice, then sugar residue was obtained from the H_2_O layer. The sugar fraction was reacted with L-cysteine methyl ester hydrochloride (in pyridine, 1 mL) in an oven (60°C, 2 h).

After concentration and drying, the residue was reacted with 1-(trimethylsilyl) imidazole (0.2 mL) in an oven (60°C, 1 h) and then extracted with n-hexane. The n-hexane fraction was acquired for GC analysis. The sugar was identified as D-glucose (*t*_R_/min) 25.170, reference D-glucose (*t*_R_/min) 25.172.

### Computational section

All calculations and processing were conducted using ORCA 5.0.4 and Python 3.10.6. The optimization of the structures used for CD and NMR calculations was performed in pyridine solvents and B3LYP-D3BJ/6–31 g (d, p) levels in the CPCM model, using tight criteria and checking for the absence of virtual frequencies. NMR calculations were performed using the SMD model and at the revTPSS/PCSSEG −1 level (10.1021/ACS.JCTC. 1C00604 indicates that this is a good level, and ECD calculations were performed using TDDFT under the SMD model. We calculated 90 excited states at the ωb97x-d4/def2-TZVP level to cover the excited levels as much as possible, and the rotor intensity and excitation wavelength were expanded using the Gauss function. The FWHM was set at 10 nm to draw the spectrum.

### Bioactivity assays

#### Luciferase reporter gene assay

The cells used in this experiment were MDA-MB-231 stable reporter cell lines transfected with ARE-luciferase plasmid in the previous study (Chen, et al., 2016). The cells were cultured in medium containing puromycin (1.5 mg mL^−1^) in 48 well plates for 24 h. The isolated constituents **1**–**4** (1–10 *μ*M) were added to the cells for 24 h. The luciferase activities were tested using the manufacturer’s protocol after the digestion of the cells. TBHQ was used as positive control.

#### Cytotoxic activity assay

The CCK8 method was applied in a cell viability assay in human MDA-MB-231 cells and human A549 cells. Compounds **1**–**4** (10–80 *μ*M) were added to the cells for 24 h. The medium was discarded, and CCK8 solution was added and then cultured for 90 min. Absorbance at 450 nm was recorded and used to calculate cell viability.

## Results and discussion

### Structure elucidation

**FIGURE 1:**
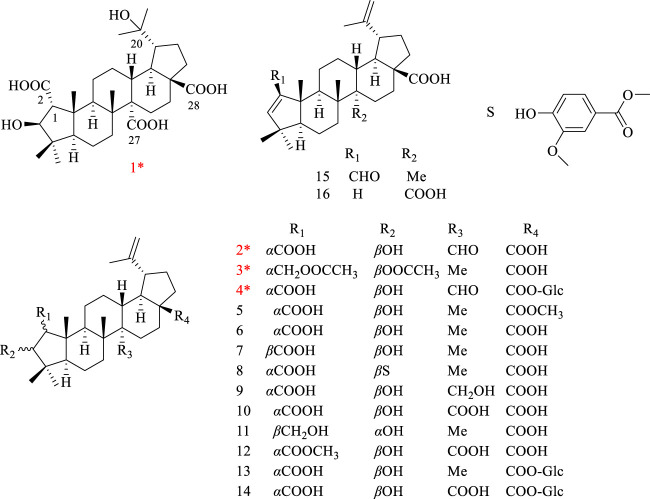
Structures of compounds **1**–**4**.

The stems of *H*. *dulcis* were extracted with 70% EtOH, and EtOAc extraction fraction was further isolated by multi-separation methods, acquiring four novel ceanothane-type triterpenoid hovendulcisic acids A-D (**1**–**4**) and twelve known: methylceanothate (**5**) (Leal, et al., 2010), ceanothic acid (**6**) (Ganapaty, et al., 2006), epiceanothic acid (**7**) (Li, et al., 2007), 3-O-vanillylceanothic acid (**8**) (Ganapaty, et al., 2006), 27-hydroxyceanothic acid (**9**) (Li, et al., 1997), ceanothetric acid (**10**) (Li, et al., 1997), ceanothanolic acid (**11**) (Lee, et al., 1997), ceanothetric acid 2-methyl ester (**12**) (Lee, et al., 1997), ceanothic acid 28β-glucosylester (**13**) (Lee, et al., 1991), hovetrichoside H (**14**) (Yoshikawa, et al., 1998), zizyberenalic acid (**15**) (Zhang, et al., 2012), and ceanothenic acid (**16**) (Jou, et al., 2004) (Figure 1).

**FIGURE 2:**
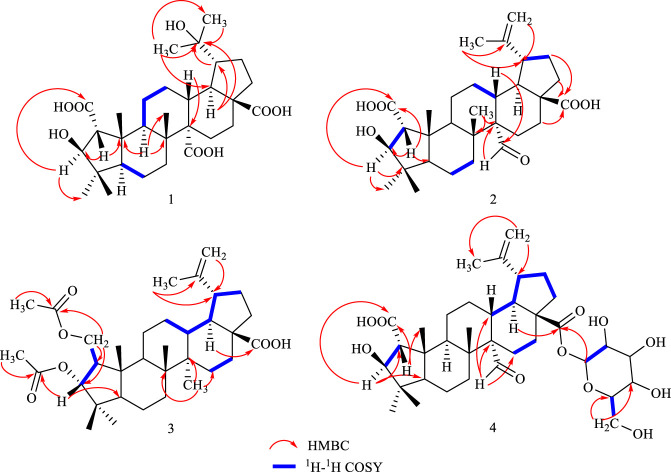
Key ^1^H−^1^H COSY and HMBC correlations in compounds **1**–**4**.

Compound **1** was a white amorphous powder, and its formula of C_30_H_45_O_8_ was deduced by HR-ESI-MS peaks [M-H]^-^ at *m/z* 533.3124 (calcd. for C_30_H_45_O_8_, 533.3109). IR data showed absorption bands for OH (3330.0 cm^−1^), C=O (1715.6, 1682.6 cm^−1^), and C=C (1644.6 cm^−1^). ^1^H NMR spectrum (Table 1) exhibited six methyl groups [*δ*_H_ 1.48(3H, s, H_3_-25), 1.30 (3H, s, H_3_-23), 1.30 (3H, s, H_3_-26), 1.25 (3H, s, H_3_-24), 1.47 (3H, s, H_3_-29), and 1.39 (3H, s, H_3_-30)]. ^13^C NMR and DEPT-135 data (Table 2) exhibited 30 carbon signals, including six methyls, eight methylenes, seven methines, and nine quaternary carbons (including three carboxylic acid carbons at *δ*_C_ 180.1, 179.2, 178.4). The hydrogen signals and relevant carbon atoms were assigned by HSQC spectrum, and the above information suggested that **1** possessed a triterpenoid skeleton. The NMR data of **1** was similar to those of the known compound ceanothetric acid but without the isoallyl signals (Yoshikawa et al., 1998b). Compared with ceanothetric acid, the molecular weight of **1** added 18 Da and the unsaturation degree decreased by 1, indicating that the double bond between C-20 and C-30 of ceanothetric acid was oxidized and a hydroxyl group was added. The correlations in the HMBC spectrum between C-20 (72.9) and H-29 (*δ*_H_ 1.47), H-30 (*δ*_H_ 1.39) revealed that **1** lost a double bond between C-20 and C-30, which was distinct from the typical ceanothane-type triterpenoid. The HMBC correlations between C-20 (*δ*_C_ 72.9) and H-18 (*δ*_H_ 2.89), H-19 (*δ*_H_ 2.25) also confirmed the position of quaternary carbon (Figure 2). Three carboxylic acid signals (*δ*_C_ 180.1, 179.2, 178.4) were attributed to C-28, C-27, and C-2 according to the HMBC spectra and literature data (Kang et al., 2017).

**FIGURE 3:**
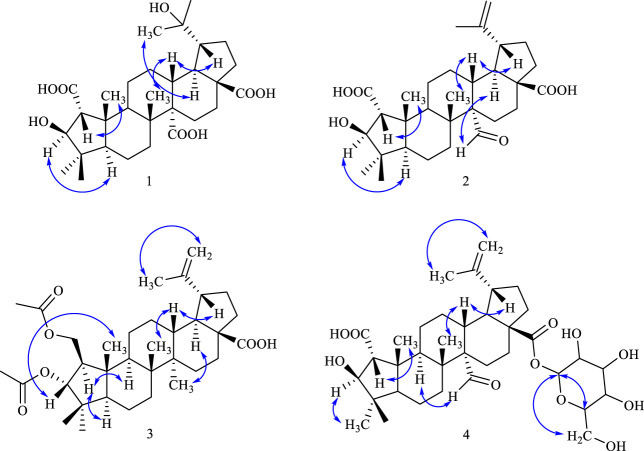
Key NOESY correlations in compounds **1**–**4**.

**FIGURE 4:**
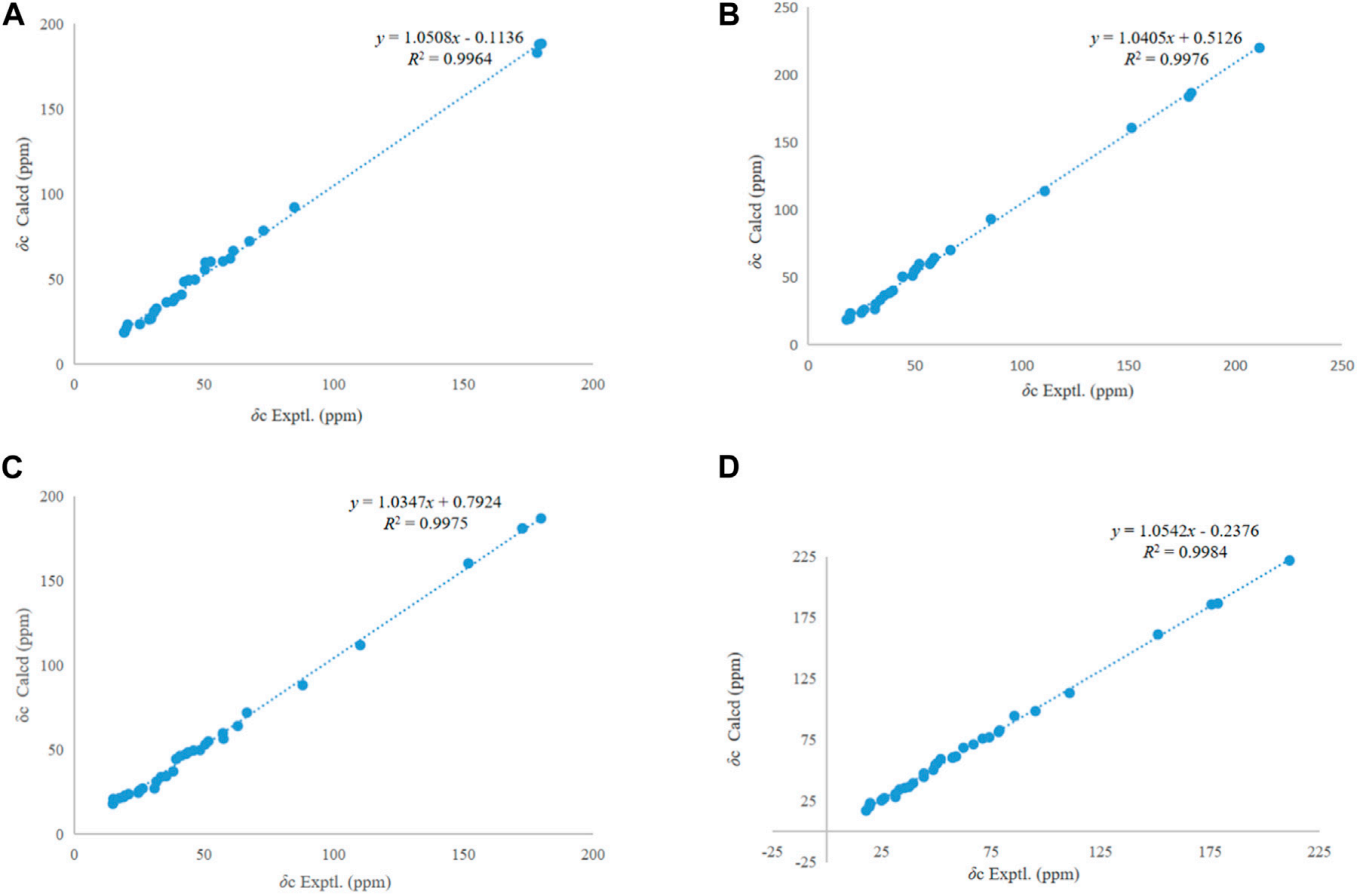
Regression analysis of experimental and calculated ^13^C NMR chemical shifts of **1**–**4** **(A–D)**.

**FIGURE 5:**
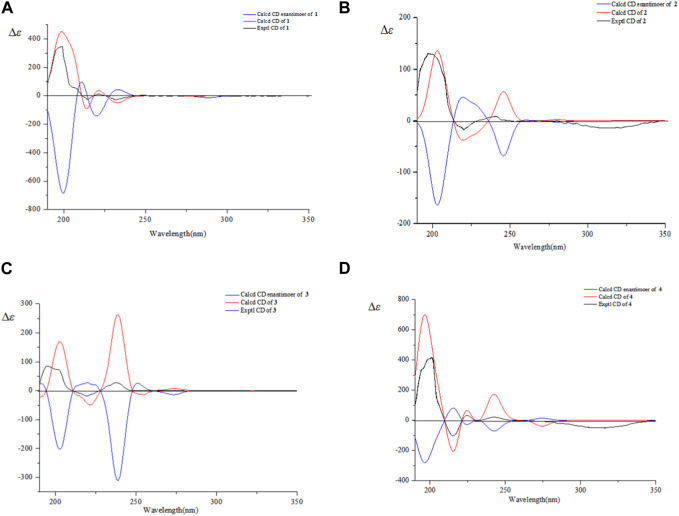
Experimental and calculated ECD spectra of **1**–**4** **(A–D)**.

NOESY correlations between H−1 (*δ*_H_ 3.30) and *δ*_H_ H-25 (1.48), H-3 (*δ*_H_ 4.87) and H-5 (*δ*_H_ 2.22) suggested the *α*-configuration of C-2 and *β*-configuration of C-3 (Li et al., 1997). NOESY correlations between *δ*_H_ 2.25 (H-19) and *δ*_H_ 3.13 (H-13) indicated *α*-configuration at C-20 (Figure 3). The ^13^C NMR chemical shift calculations for the 1*S*, 5*R*, 7*R*, and 23*R* of **1** agreed well with the experimental data, and the correlation coefficient R^2^ was 99.64% (Figure 4A). Finally, the absolute configuration of **1** (1*S*, 5*R*, 7*R*, and 23*R*) was determined according to the comparison experimental data with calculated ECD spectra (Figure 5A).

Taken together, compound **1** was novel and was identified as 2*α*-carboxy-3*β*, 20-dihydroxy-A (1)-norlup-27, 28-dioic acid and named “hovendulcisic acid A”.

Compound **2** was obtained as a white amorphous powder, and the formula was observed to be C_30_H_44_O_6_ according to HR-ESI-MS data [M-H]^-^ at *m/z* 499.3060 (calcd for C_30_H_43_O_6_, 499.3054). The IR absorption bands exhibited OH (3475.3 cm^−1^), C=O (1722.0, 1718.9, 1688.0 cm^−1^), C=C (1644.6 cm^−1^), and C-O (1235.4 cm^−1^). The ^1^H and ^13^C NMR spectral data of **2** are similar to those of ceanothetric acid, but added a aldehyde group instead of carboxylic acid carbons (Yoshikawa et al., 1998). The correlations in the HMBC spectrum between C-2 (*δ*_C_ 178.2) and H−1 (*δ*_H_ 2.50), H-3 (*δ*_H_ 4.08), C-28 (*δ*_C_ 179.4), and H-16 (*δ*_H_ 1.59), H-21 (*δ*_H_ 1.38), H-22 (*δ*_H_ 1.90) confirmed the position of the carboxylic acid groups. The HMBC correlations between C-2 (*δ*_C_ 178.2), C-4 (*δ*_C_ 44.2), and H-3 (*δ*_H_ 4.08) also indicated that the hydroxy was located at C-3. The HMBC correlations between the C-14 (*δ*_C_ 59.0) and aldehyde proton signal H-27 (*δ*_H_ 10.13), C-27 (*δ*_C_ 211.3) and H-13 (*δ*_H_ 2.53), H-15 (1.92) indicated that the aldehyde group was located at C-27 (Figure 2). NOESY correlations between *δ*_H_ 1.55 (H-18) and *δ*_H_ 10.13 (H-27) suggested that the *α*-configuration was at C-27 (Figure 3). The ^13^C NMR chemical shift calculations for the 1*S*, 5*R*, 7*R*, and 23*R* of **2** correlated well with the experimental result, and the correlation coefficient R^2^ was 99.76% (Figure 4B). The absolute configuration of **2** was determined by ECD method (Figure 5B). Taken together, compound **2** was identified as 2*α*-carboxy-3*β*-hydroxy-A (1)-norlup-20 (29)-en-27-aldehydo-28-oic acid and named “hovendulcisic acid B”.

Compound **3** was obtained as a white amorphous powder, and its formula was C_34_H_52_O_6_ though HR-ESI-MS data [M-H]^-^ at *m/z* 555.3699 (calcd for C_34_H_51_O_6_, 555.3680). The IR data exhibited the existence of OH (3397.0 cm^−1^), C=O (1739.6, 1701.3 cm^−1^), C=C (1644.6 cm^−1^), and C-O (1186.2, 1029.0 cm^−1^). The ^13^C NMR and DEPT spectra exhibited 34 carbon resonances, including eight methyls, ten methylenes (including an oxygenated methylene group at *δ*_C_ 66.56), seven methines (including an oxygenated methine group at *δ*_C_ 88.07), and nine quaternary carbons (including a carboxylic acid carbons at *δ*_C_ 179.91 and two ester groups at *δ*_C_ 172.82, 172.70). The 1D NMR data of **3** were similar to those of **2** but different from the existence of two oxygenated methylene protons [*δ*_H_ 4.47 (1H, dd, *J* = 11.5, 4.5 Hz, H-2*α*), 4.04 (1H, dd, *J* = 11.5, 7.8 Hz, H-2*β*)] and one oxygenated methine proton [*δ*_H_ 4.94(1H, d, *J* = 8.7 Hz, H-3)] (Tables 1 and 2). The above data indicated that **3** was a ceanothane-type triterpenoid with two acetyl groups. HMBC correlation of oxygenated methylene proton [*δ*_H_ 4.47, 4.04 (H-2)] with *δ*_C_ 172.8 (C-1′) indicated that one acetyl group was linked at the C-2. HMBC correlation between oxygenated methine proton *δ*_H_ 4.94 (H-3) and *δ*_C_ 172.7 (C-3′), suggesting that another acetyl group was located at C-3 (Figure 2). According to the correlations in the HMBC spectrum between C-1’ (*δ*_C_ 172.8) and H-2 ′ (*δ*_H_ 2.05), C-3’ (*δ*_C_ 172.7) and H-4’ (*δ*_H_ 1.98), the position of the methyl proton can be assigned. The position of the isopropenyl group can be determined according to the HMBC correlations between C-19 (*δ*_C_ 48.6), C-20 (*δ*_C_ 151.9), and [*δ*_H_ 4.73, 4.61 (H-29)], H-30 (*δ*_H_ 1.71). NOESY correlations between *δ*_H_ 1.86 (H−1) and *δ*_H_ 1.22 (H-5), *δ*_H_ 1.69 (H-9), between *δ*_H_ 4.94 (H-3) and *δ*_H_ 0.89 (H-25) indicated the *β*-configuration of C-2 and *α*-configuration of C-3’ (Figure 3).

The ^13^C NMR chemical shift calculations confirmed the relative configuration of **3** (Figure 4C) while the experimental and calculated ECD data confirmed its absolute configuration (Figure 5C). Therefore, compound **3** was identified as 2*β*, 3*α*-diacetyl-A (1)-norlup-20 (29)-en-28- oic acid and named “hovendulcisic acid C”.

Compound **4** was isolated as a white amorphous powder with formula C_36_H_54_O_11_ according to HR-ESI-MS data [M-H]^-^ at *m/z* 661.3597 (calcd for C_36_H_53_O_11_, 661.3582). IR absorption bands exhibited the existence of OH (3391.4 cm^−1^), C=O (1748.0, 1722.0, 1699.0 cm^−1^), C=C (1641.4 cm^−1^) ,and C-O (1065.5, 1028.0 cm^−1^). The NMR data of **4** were similar to those of **2** but added a sugar residue [*δ*_H_ 5.50 (1H, d, *J* = 8.0 Hz, H-1′ in Glu), proton signals at *δ*_H_ 3.32–3.82] (Tables 1 and 2). The sugar residue was finally identified as D-glucose according to acid hydrolysis and GC analysis. HMBC correlations between C-28 (*δ*_C_ 175.7) and H-1′ in Glu (*δ*_H_ 5.50) indicated that the glycosidic bonds were located at C-28. The HMBC correlations between the aldehyde proton signal H-27 (*δ*_H_ 10.12) and C-14 (*δ*_C_ 58.9), C-13 (*δ*_C_ 39.3), C-15 (*δ*_C_ 25.4) indicated that the aldehyde group was located at C-27. The position of the isopropenyl group can be determined according to the HMBC correlations between [*δ*_H_ 4.74, 4.62 (H-29)] and C-19 (*δ*_C_ 48.6), C-30 (*δ*_C_ 19.3).

The relative configuration of **4** was deduced by the experimental and calculated ^13^C NMR method, while the absolute configuration of **4** was approved by experimental and calculated ECD data (Figures 4D, 5D). Therefore, **4** was elucidated as 2*α*-carboxy-3*β*-hydroxy-A (1)-norlup-20 (29)-en-27-aldehydo-28-oic acid (hovendulcisic acid B) 28-*β*-D-glucopyranoside and named “hovendulcisic acid D”.

### Putative biosynthetic pathways analysis

**SCHEME 1:**
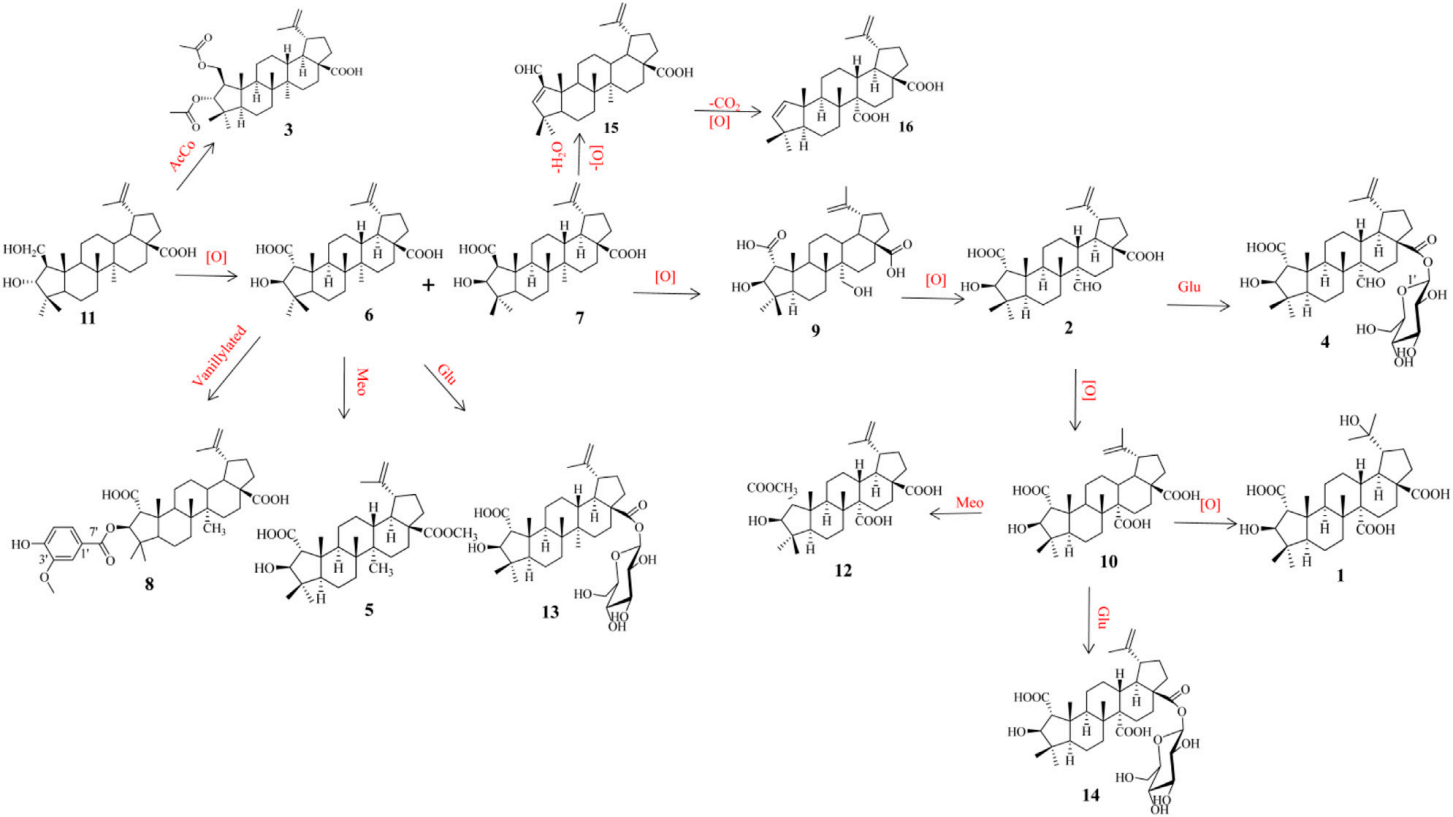
Putative biosynthetic pathways to **1**–**16**.

The plausible biosynthetic pathway of compounds **1**–**16** is shown in Scheme 1. The precursor ceanothanolic acid (**11**), which was first obtained from *Paliurus hemsleyanus* (Lee, et al., 1997), was oxidated at C-2 to give ceanothic acid (**6**) and epiceanothic acid (**7**), diacetylated at C-2 and C-3 to afford hovendulcisic acid C (**3**). Then ceanothic acid (**6**) was vanillylated with OH at C-3 to give 3-*O*-vanillylceanothic acid (**8**), while ceanothic acid (**6**) was methyl esterificated and glycosylated of C-28 to afford methylceanothate (**5**) and ceanothic acid 28*β*-glucosylester (**13**), respectively. Epiceanothic acid (**7**) was oxidated to 27-hydroxyceanothic acid (**9**), hovendulcisic acid B (**2**), ceanothetric acid (**10**), and hovendulcisic acids A (**1**) with different levels. Hovendulcisic acid B (**2**) and ceanothetric acid (**10**) were further glycosylated to hovendulcisic acids D (**4**) and hovetrichoside H (**14**), respectively. Epiceanothic acid (**7**) was dehydrated and reduced to zizyberenalic acid (**15**), which was further decarboxylated and oxygenated to ceanothenic acid (**16**).

### Bioactivity results

**FIGURE 6:**
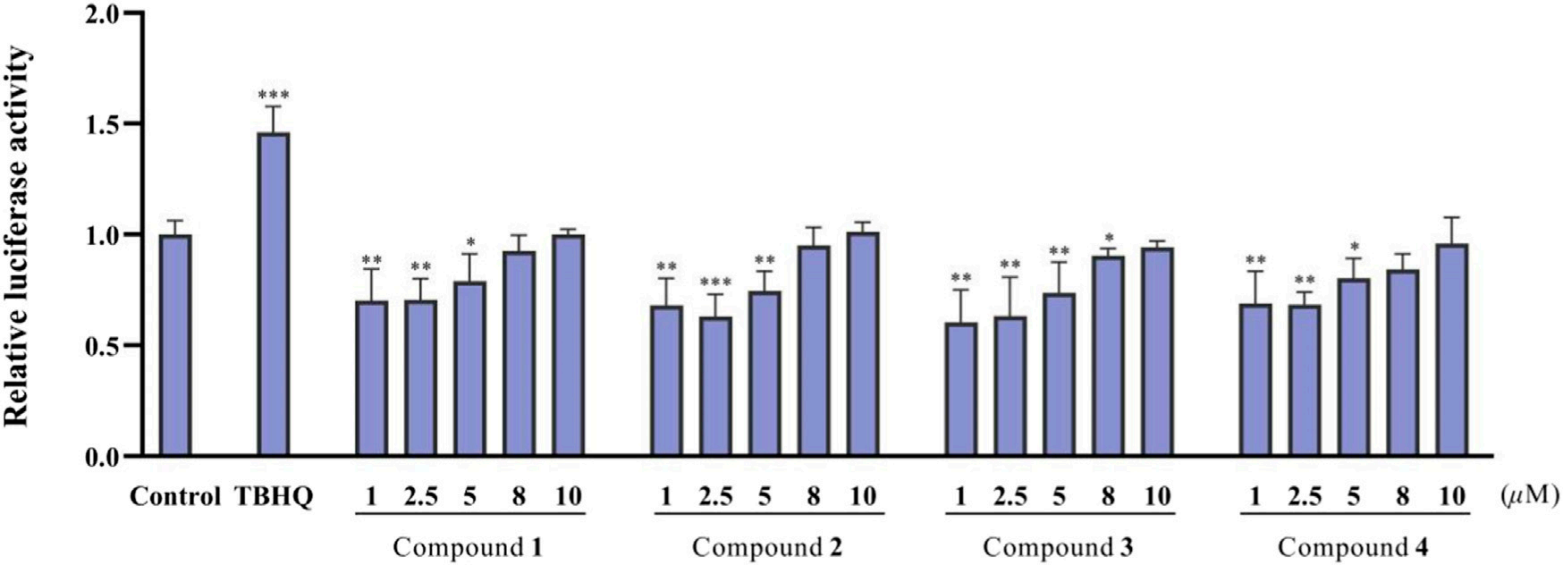
ARE-dependent luciferase activity of the isolated compounds **1**–**4** (n = 3).

**FIGURE 7:**
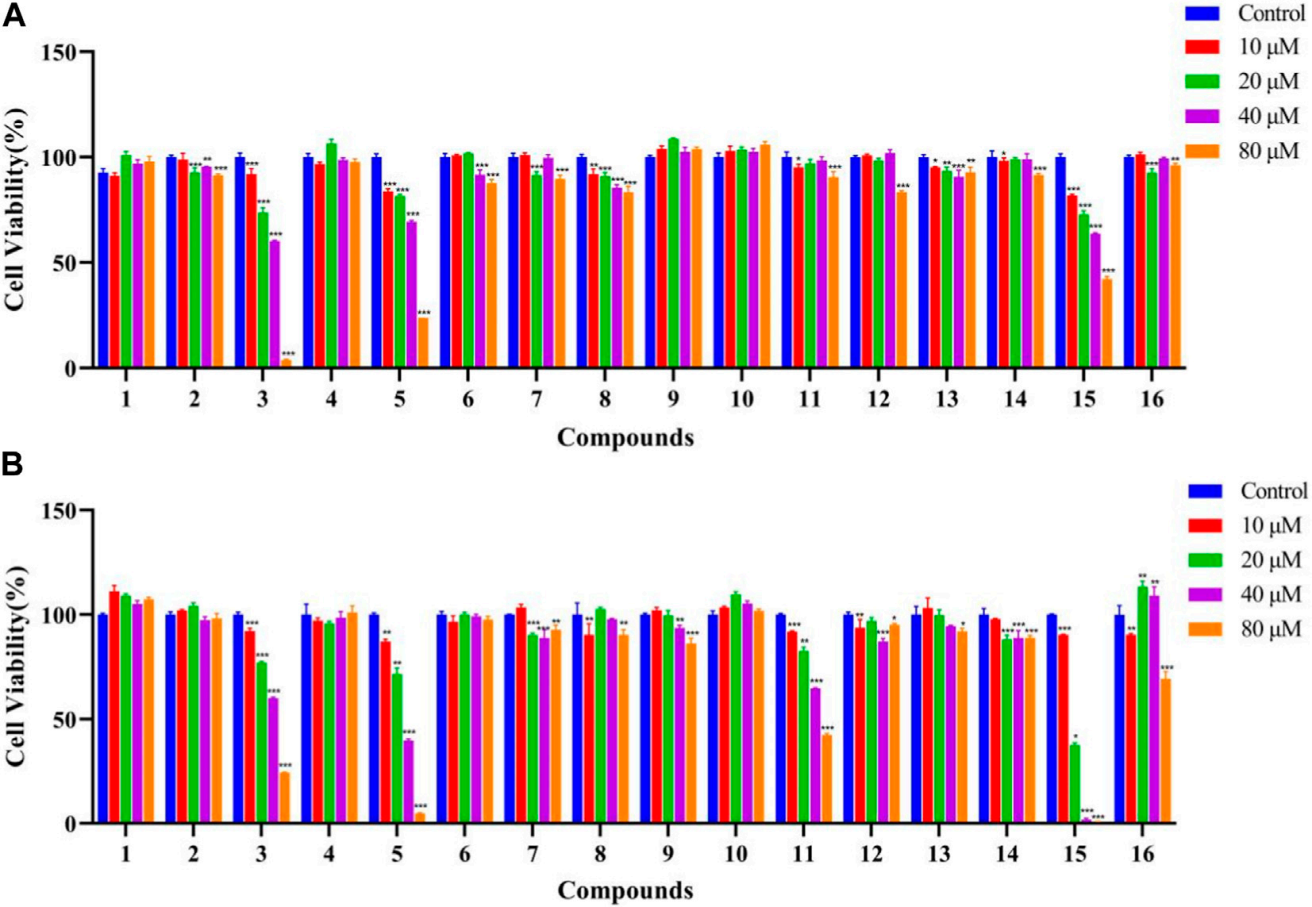
Effects of compounds **1**–**16** against A549 cells **(A)** and MDA-MB-231 cells **(B)** (*n* = 3). The data represent the mean ± SD of three experiments. *p < 0.05 and **p < 0.01 vs. the control group.

The antitumor effects of isolated compounds **1**–**16** were investigated. The results of the ARE luciferase reporter gene showed that **1**–**4** showed strong inhibitory effect at low concentrations of 1 to 5 μM (Figure 6). Generally, MDA-MB-231 cells appeared to be more sensitive than A549 cells. Compounds **3**, **5,** and **15** exhibited significant inhibitory activity against A549 cells (Figure 7A), and **3**, **5**, **12,** and **15** exhibited significant inhibitory activity against MDA-MB-231 cells (Figure 7B). The structure–activity relationships analysis revealed that the acetyl and aldehyde groups played an important role in anti-tumor activity. The above results indicated that **3** suppressed tumor activity by inhibiting Nrf2 expression.

## Data Availability

The raw data supporting the conclusion of this article will be made available by the authors, without undue reservation.
