# Supplementary material for: Hovendulcisic acid A-D: four novel ceanothane-type triterpenoids from Hovenia dulcis stems with anticancer properties
Source: Front Chem. 2024 May 14;12:1383886. doi: 10.3389/fchem.2024.1383886 (PMC11130496; doi:10.3389/fchem.2024.1383886)
Supplement: Supplementary file 1 [file DataSheet1.docx]

Figure. S1 UV spectrum of **1**

Figure. S2 IR spectrum of **1**

Figure. S3 HR-ESI-MS of **1**

Figure. S4 ^1^H NMR spectrum of **1**

Figure. S5 ^13^C NMR spectrum of **1**

Figure. S6 DEPT-135 spectrum of **1**

Figure. S7 ^1^H-^1^H COSY spectrum of **1**

Figure. S8 HSQC spectrum of **1**

Figure. S9 HMBC spectrum of **1**

Figure. S10 NOESY spectrum of **1**

Figure S11 UV spectrum of **2**

Figure. S12 IR spectrum of **2**

Figure. S13 HR-ESI-MS of **2**

Figure. S14 ^1^H NMR spectrum of **2**

Figure. S15 ^13^C NMR spectrum of **2**

Figure. S16 DEPT-135 spectrum of **2**

Figure. S17 ^1^H-^1^H COSY spectrum of **2**

Figure. S18 HSQC spectrum of **2**

Figure. S19 HMBC spectrum of **2**

Figure. S20 NOESY spectrum of **2**

Figure. S21 UV spectrum of **3**

Figure. S22 IR spectrum of **3**

Figure. S23 HR-ESI-MS of **3**

Figure. S24 ^1^H NMR spectrum of **3**

Figure. S25 ^13^C NMR spectrum of **3**

Figure. S26 DEPT-135 spectrum of **3**

Figure. S27 ^1^H-^1^H COSY spectrum of **3**

Figure. S28 HSQC spectrum of **3**

Figure. S29 HMBC spectrum of **3**

Figure. S30 NOESY spectrum of **3**

Figure. S31 UV spectrum of **4**

Figure. S32 IR spectrum of **4**

Figure. S33 HR-ESI-MS of **4**

Figure. S34 ^1^H NMR spectrum of **4**

Figure. S35 ^13^C NMR spectrum of **4**

Figure. S36 DEPT-135 spectrum of **4**

Figure. S37 ^1^H-^1^H COSY spectrum of **4**

Figure. S38 HSQC spectrum of **4**

Figure. S39 HMBC spectrum of **4**

Figure. S40 NOESY spectrum of **4**

Figure. S1 UV spectrum of **1**

Figure. S2 IR spectrum of **1**

Figure. S3 HR-ESI-MS of **1**


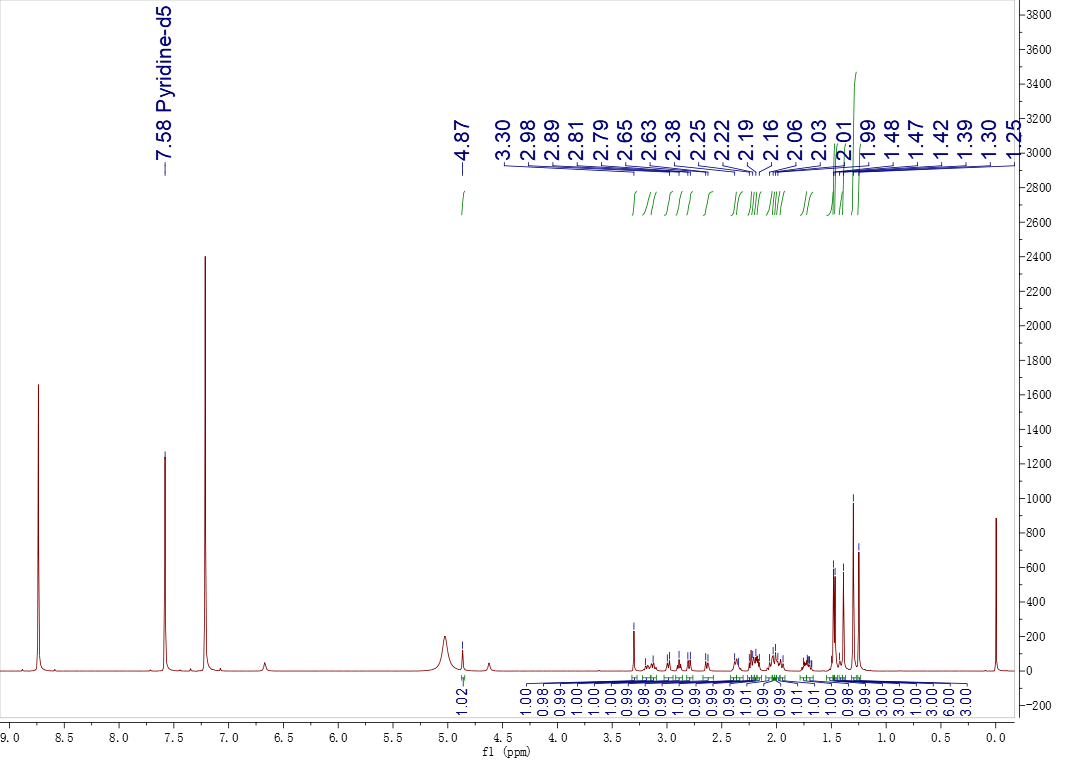


Figure. S4 ^1^H NMR spectrum of **1**


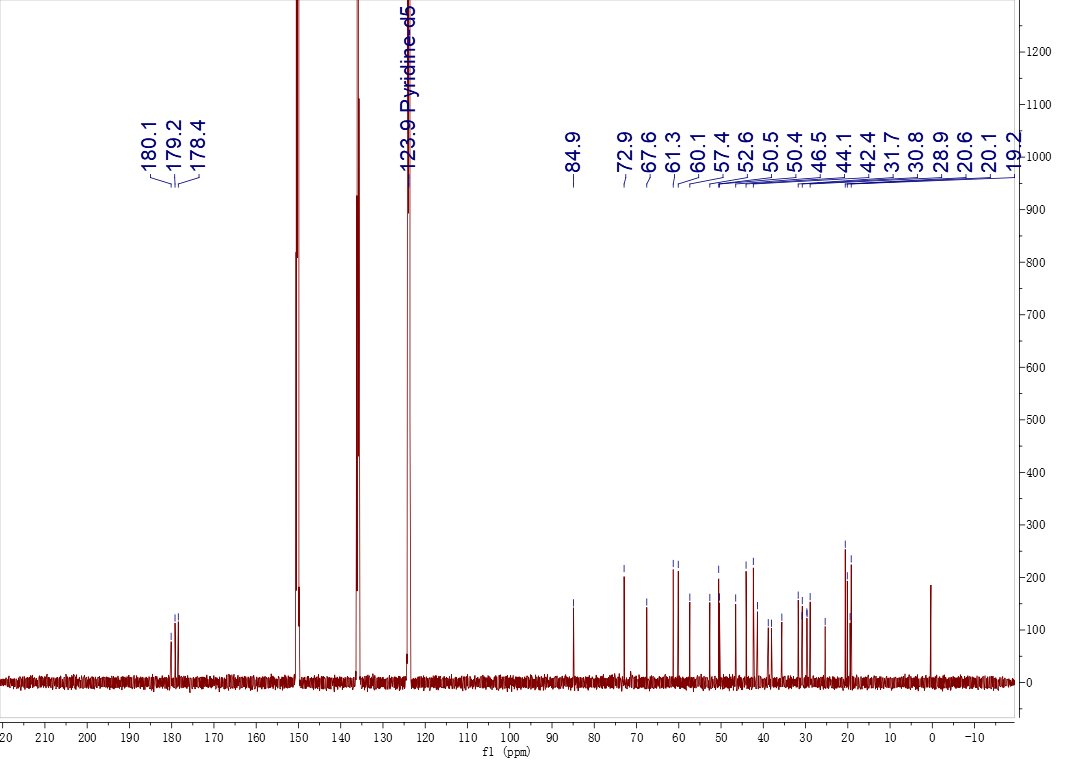


Figure. S5 ^13^C NMR spectrum of **1**


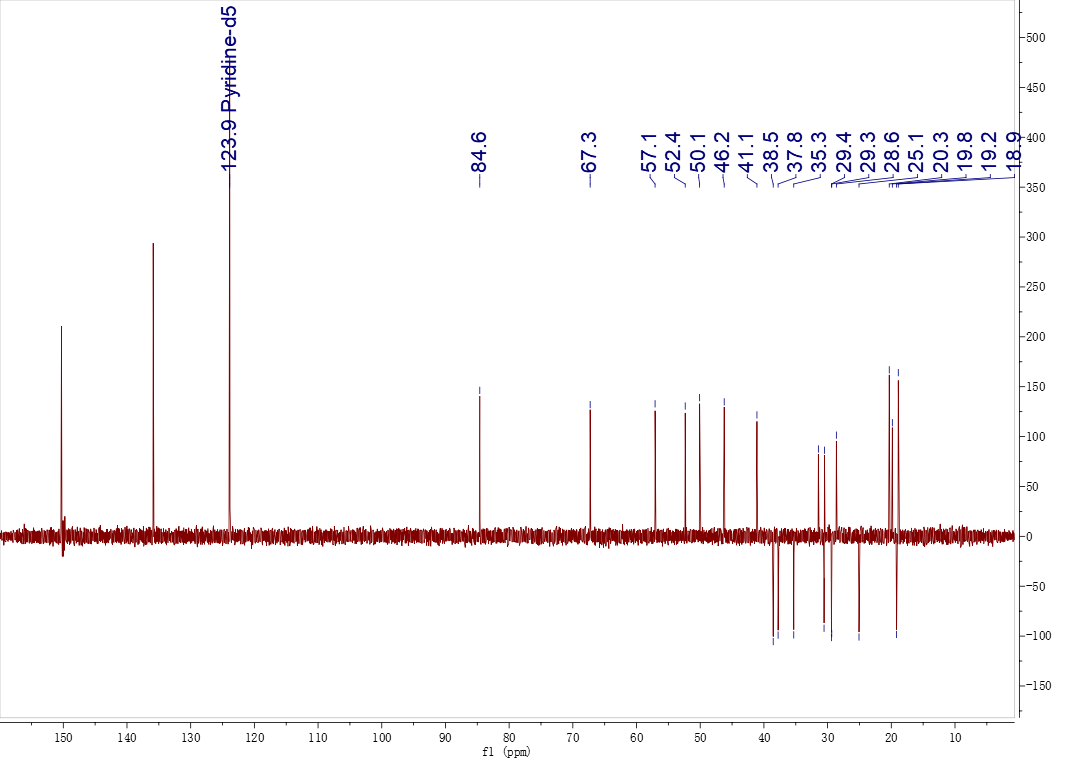


Figure. S6 DEPT-135 spectrum of **1**


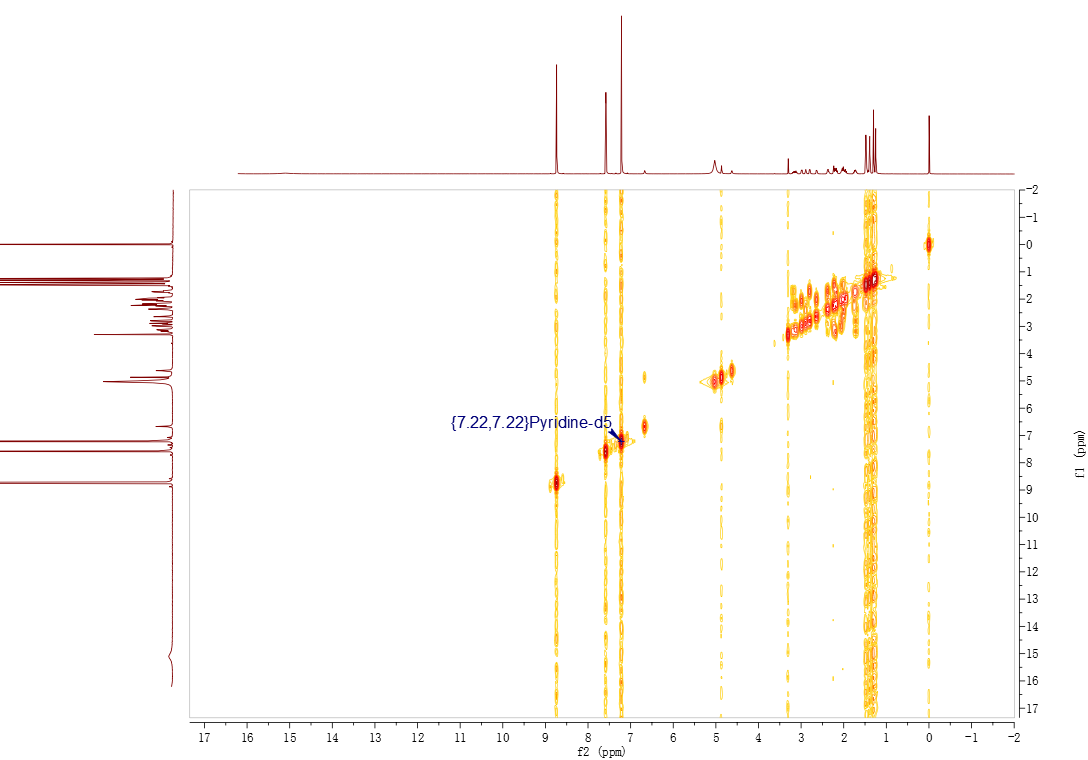


Figure. S7 ^1^H-^1^H COSY spectrum of **1**


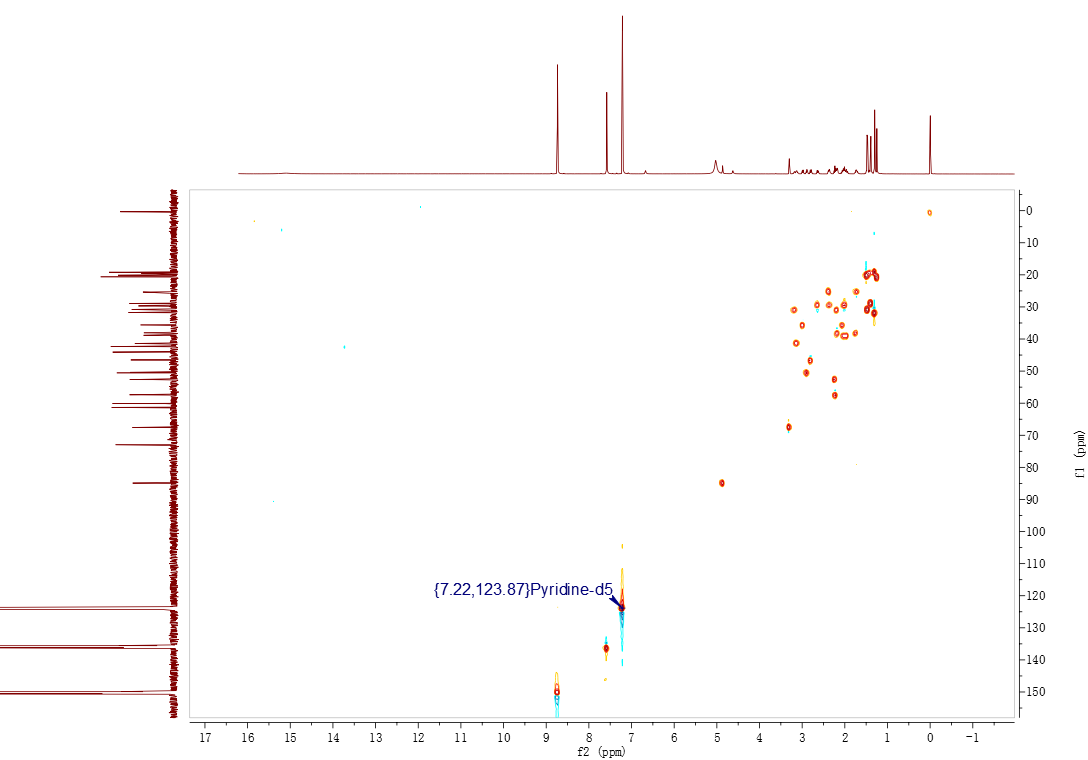


Figure. S8 HSQC spectrum of **1**


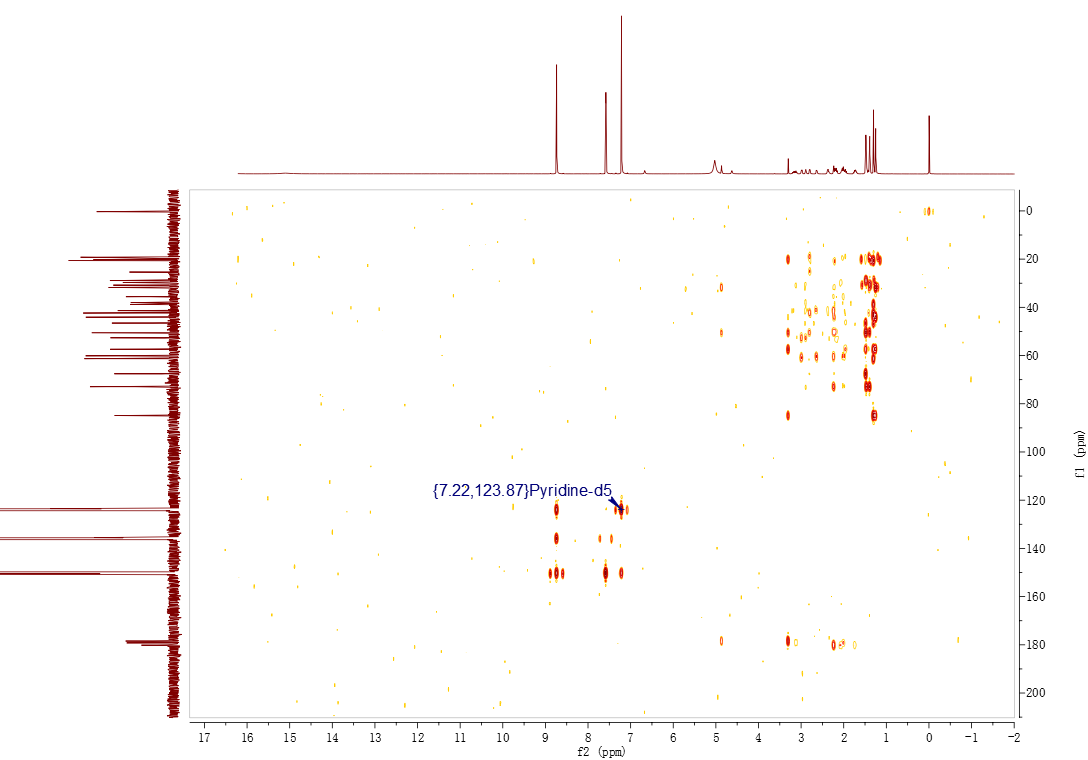


Figure. S9 HMBC spectrum of **1**


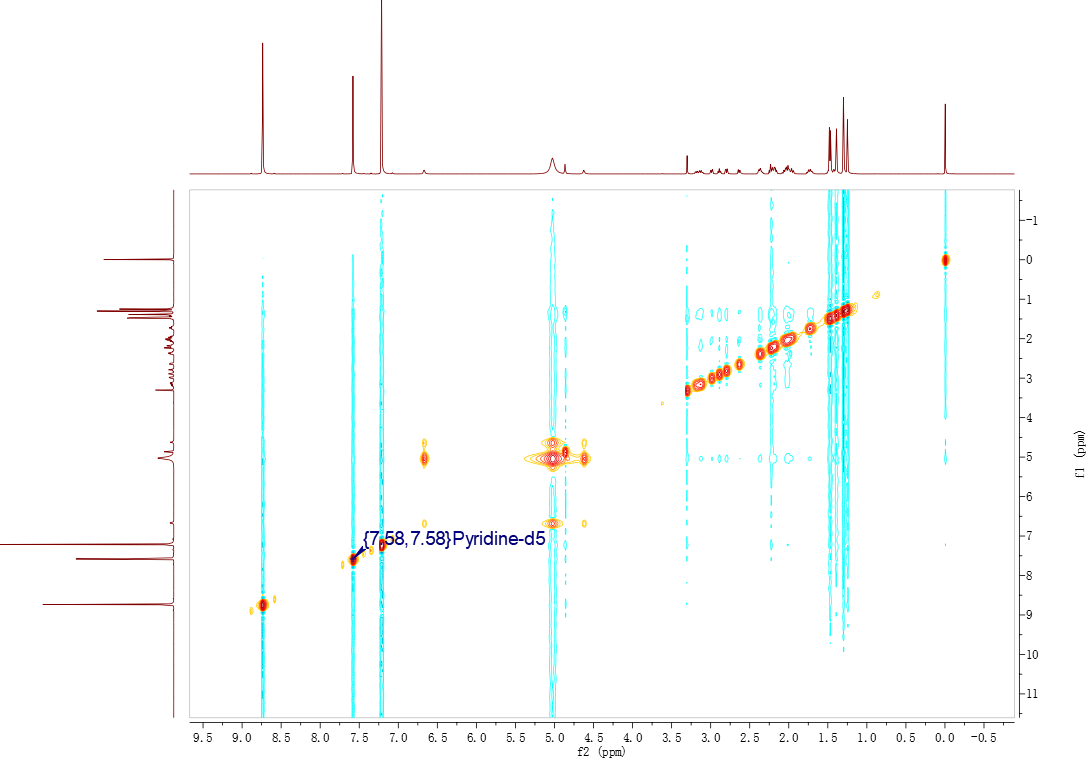


Figure. S10 NOESY spectrum of **1**

Figure S11 UV spectrum of **2**

Figure. S12 IR spectrum of **2**

Figure. S13 HR-ESI-MS of **2**


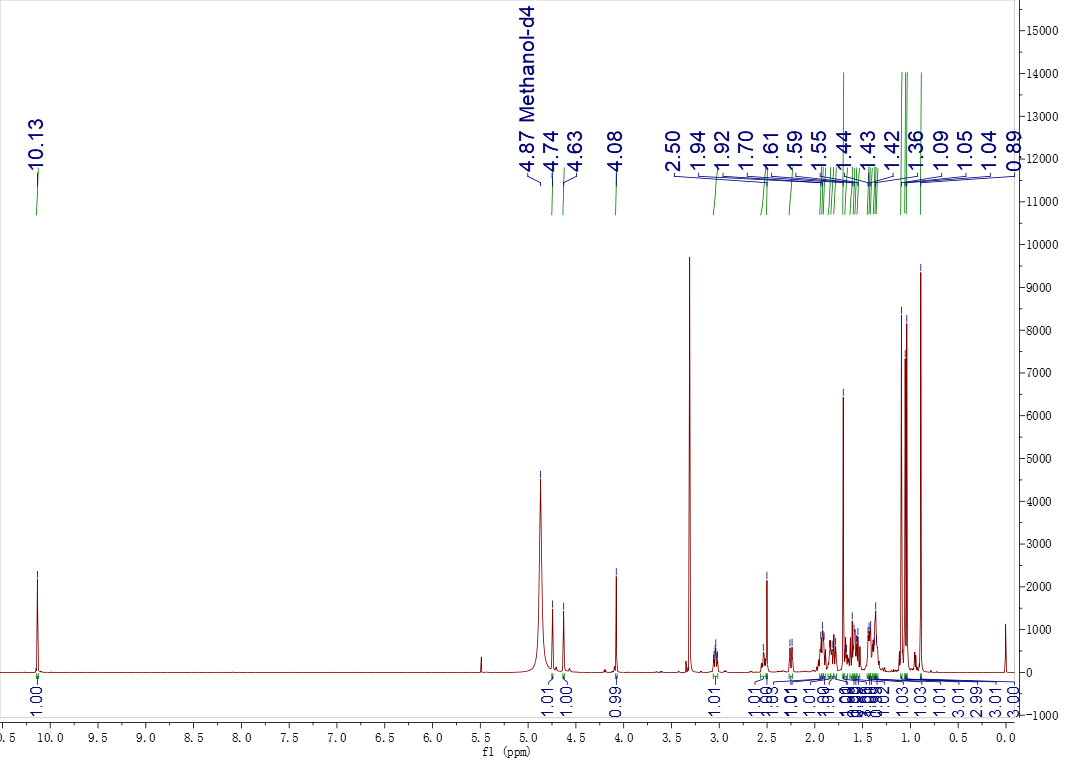


Figure. S14 ^1^H NMR spectrum of **2**


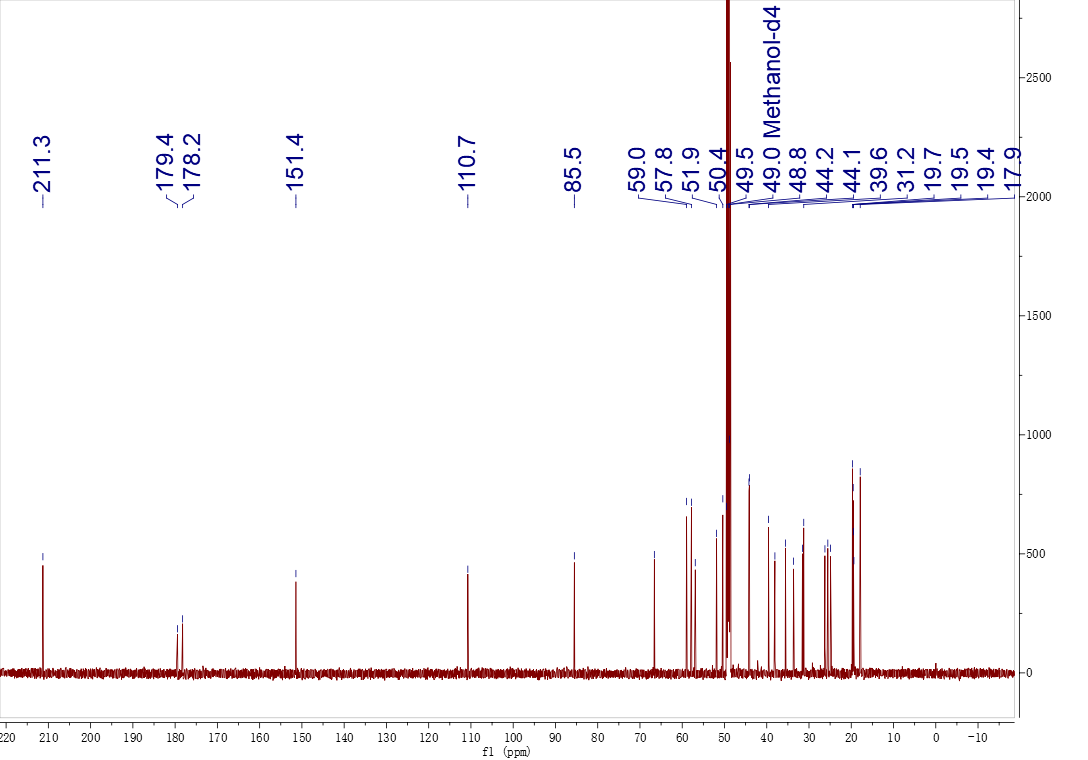


Figure. S15 ^13^C NMR spectrum of **2**


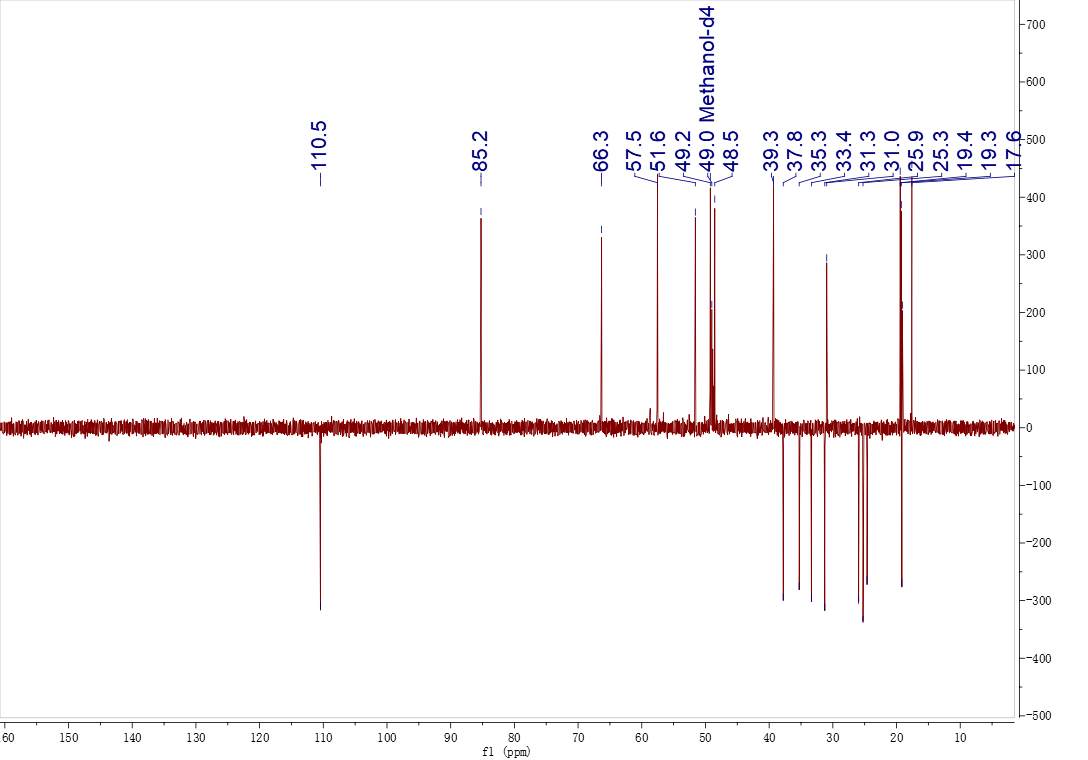


Figure. S16 DEPT-135 spectrum of **2**


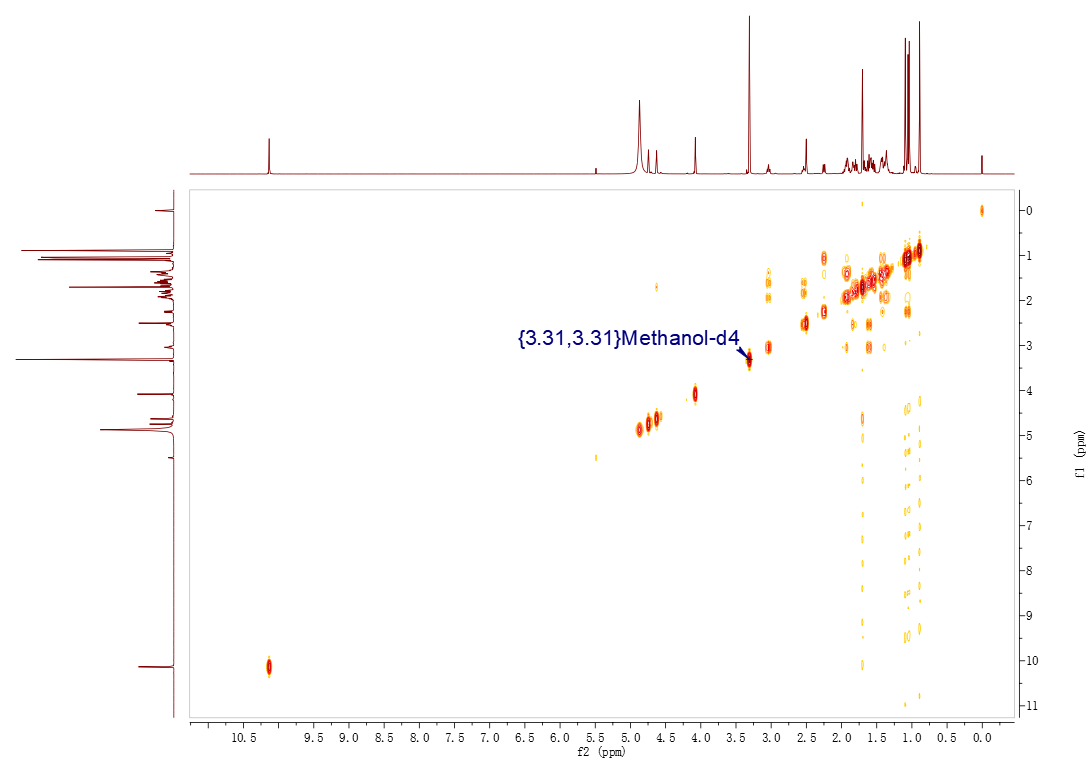


Figure. S17 ^1^H-^1^H COSY spectrum of **2**


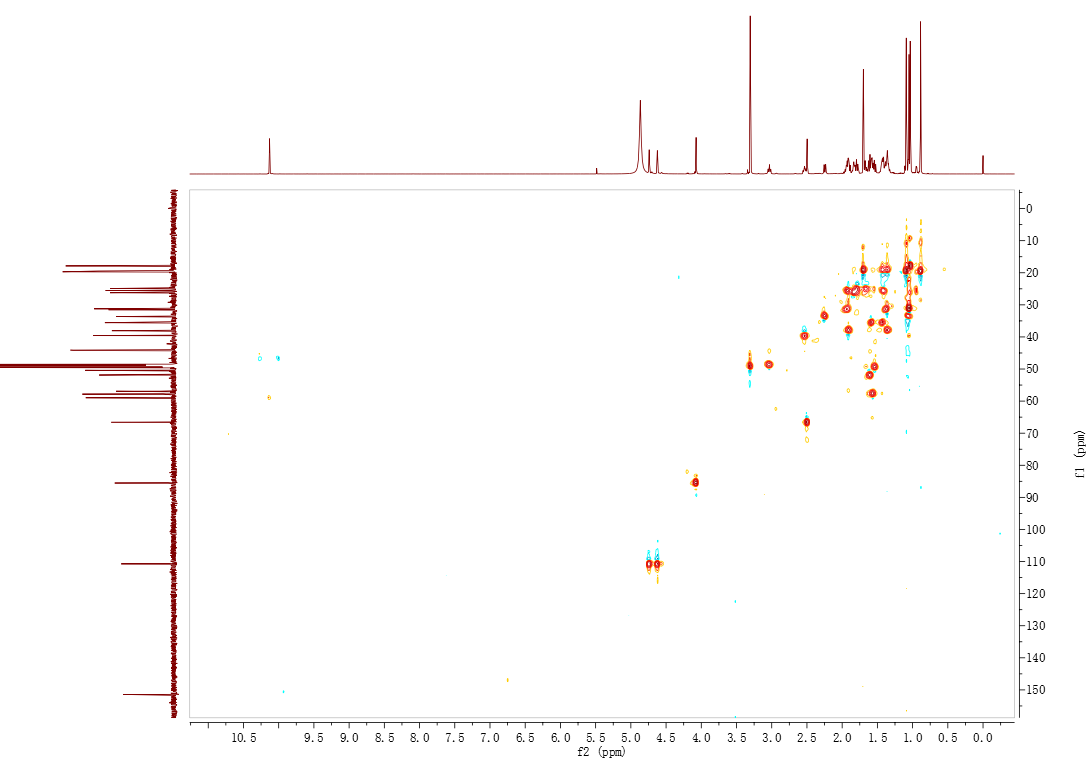


Figure. S18 HSQC spectrum of **2**


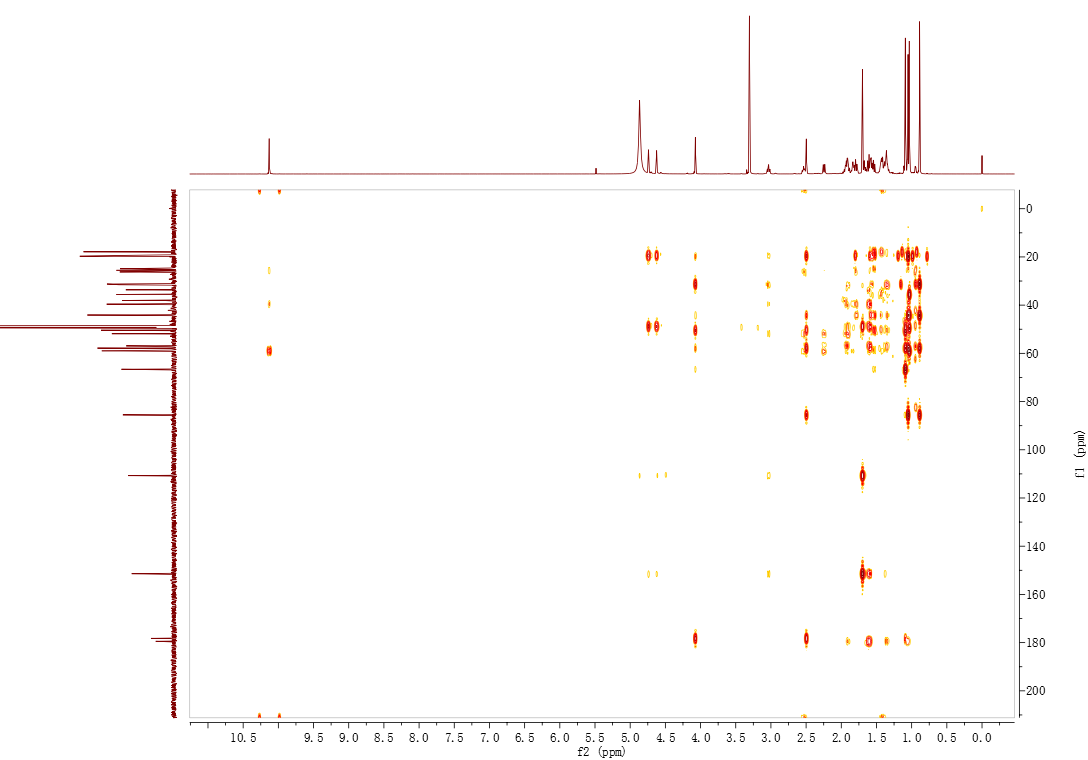


Figure. S19 HMBC spectrum of **2**


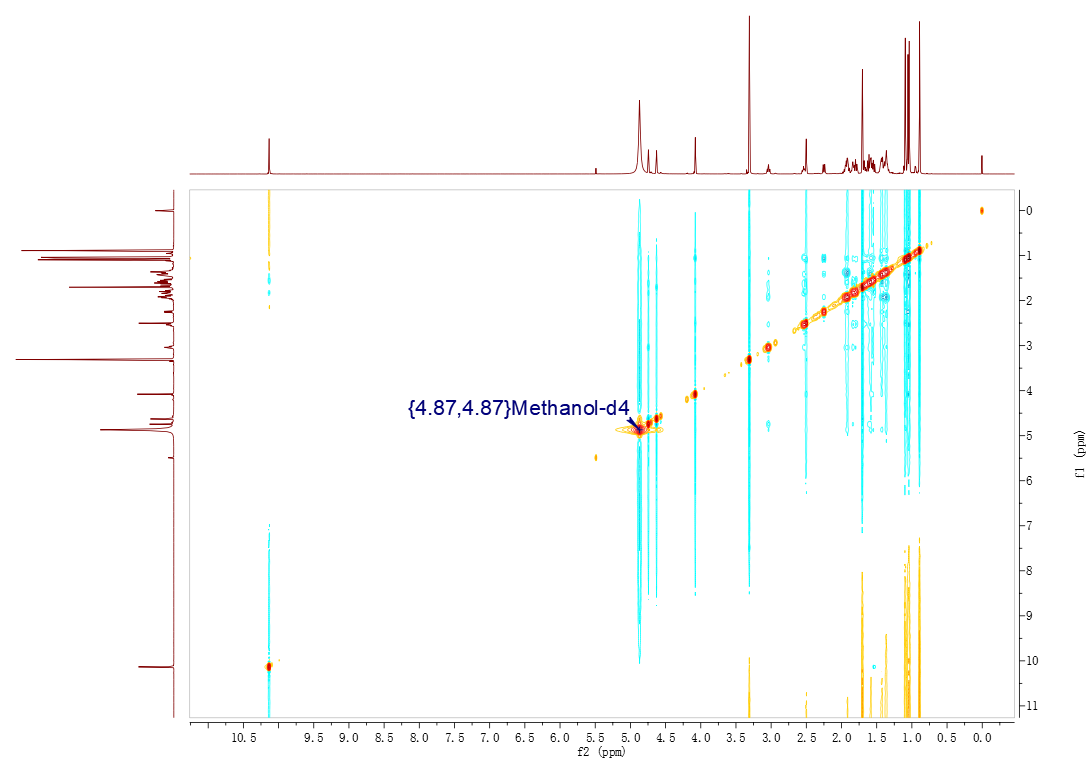


Figure. S20 NOESY spectrum of **2**

Figure. S21 UV spectrum of **3**

Figure. S22 IR spectrum of **3**

Figure. S23 HR-ESI-MS of **3**


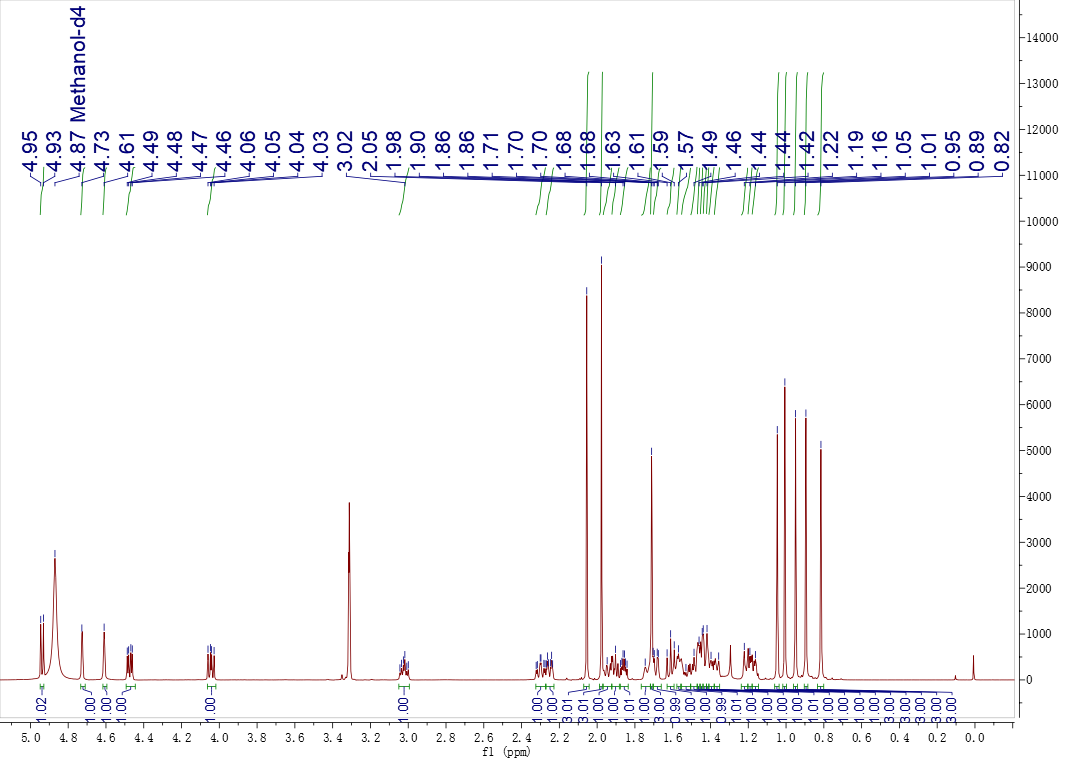


Figure. S24 ^1^H NMR spectrum of **3**


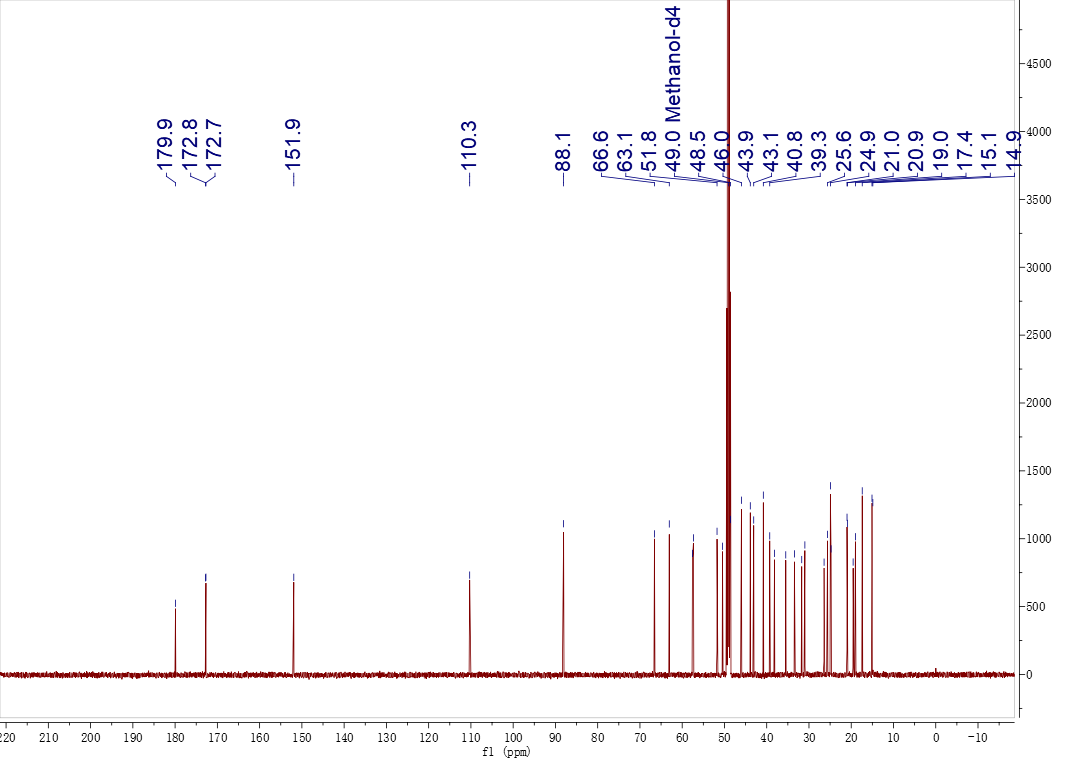


Figure. S25 ^13^C NMR spectrum of **3**


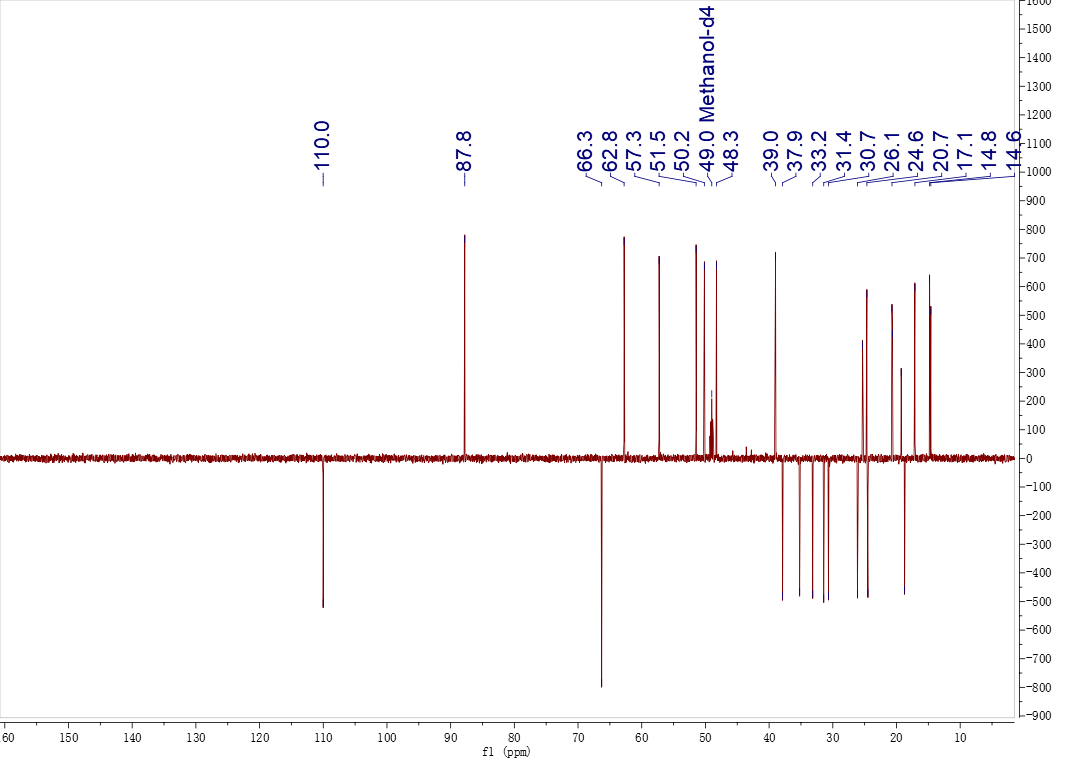


Figure. S26 DEPT-135 spectrum of **3**


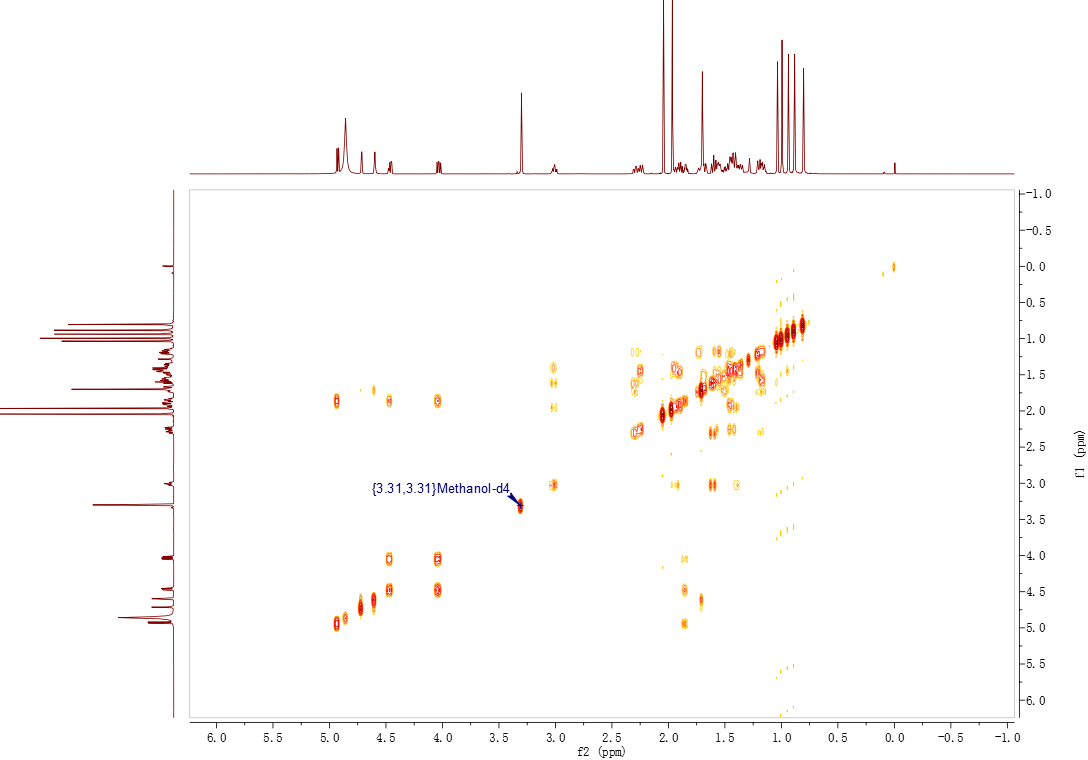


Figure. S27 ^1^H-^1^H COSY spectrum of **3**


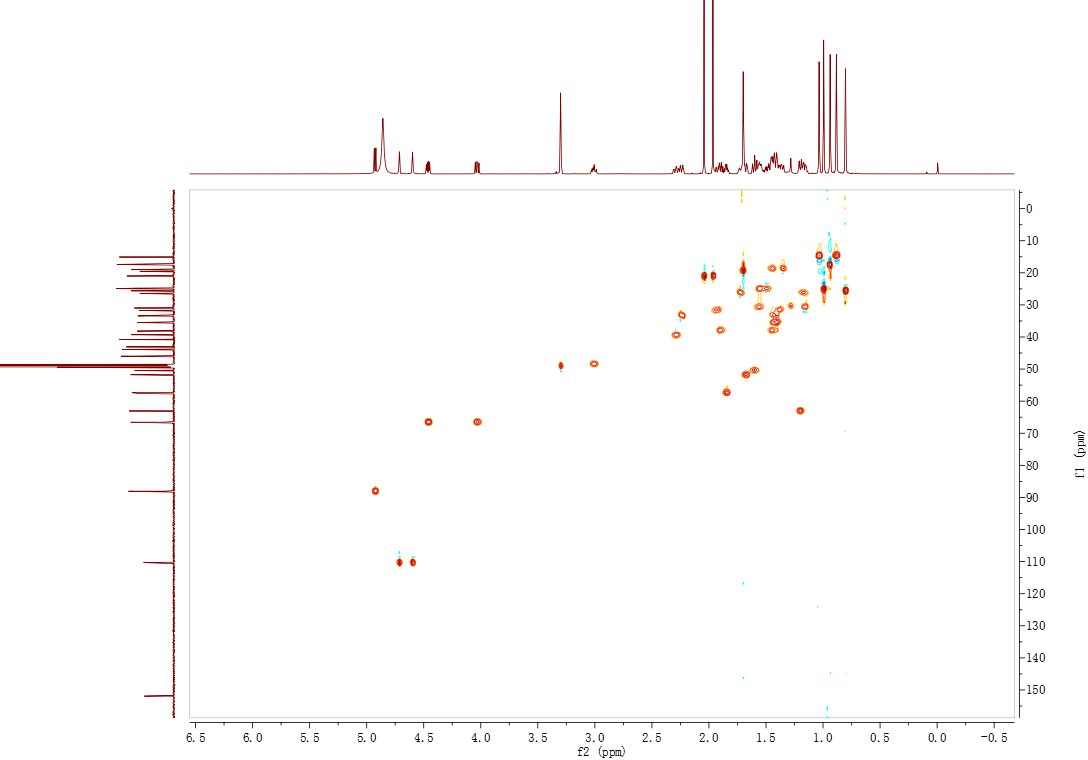


Figure. S28 HSQC spectrum of **3**


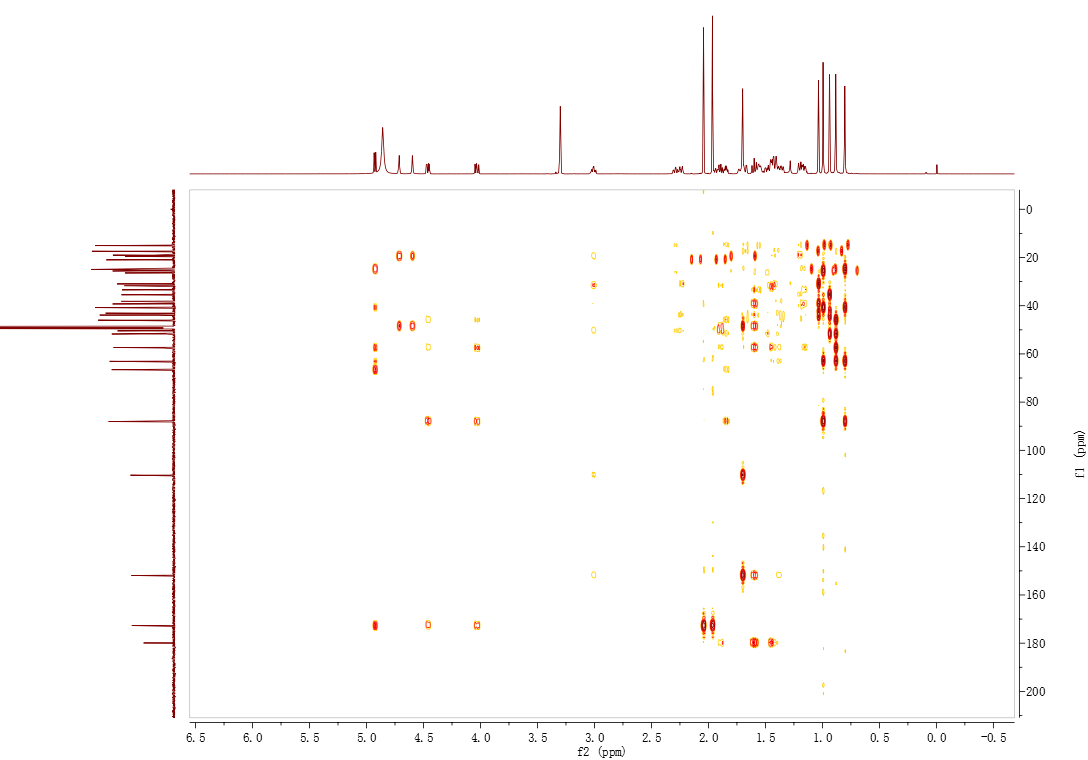


Figure. S29 HMBC spectrum of **3**


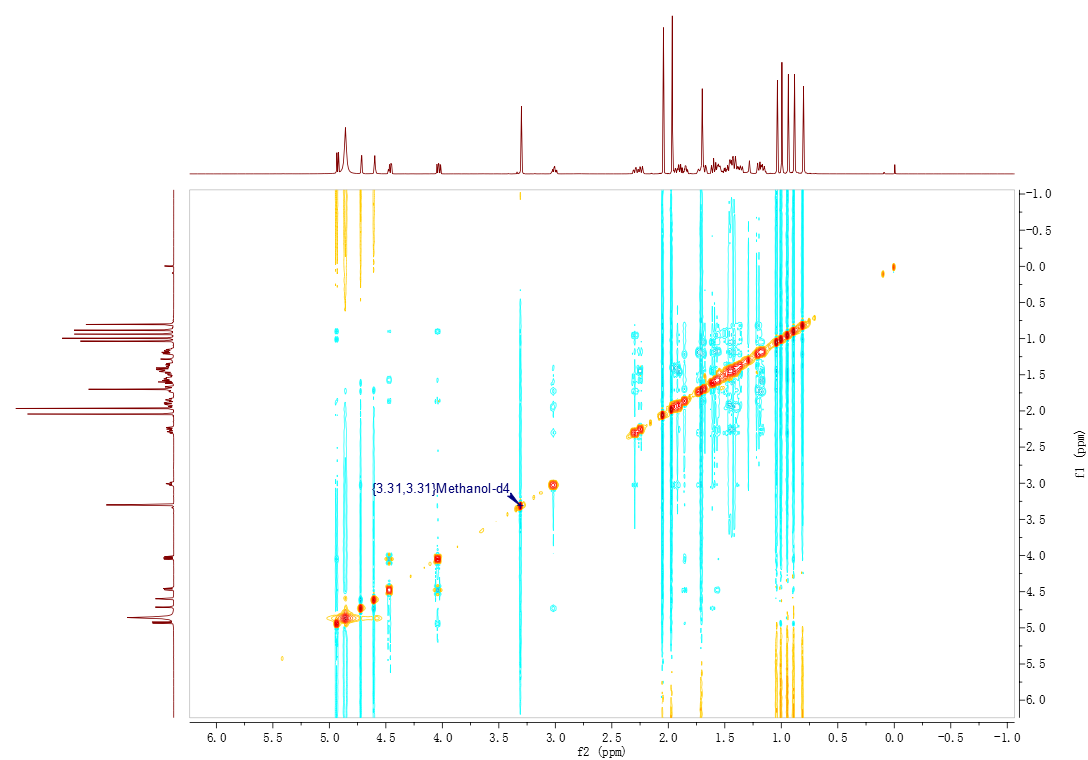


Figure. S30 NOESY spectrum of **3**

Figure. S31 UV spectrum of **4**

Figure. S32 IR spectrum of **4**

Figure. S33 HR-ESI-MS of **4**


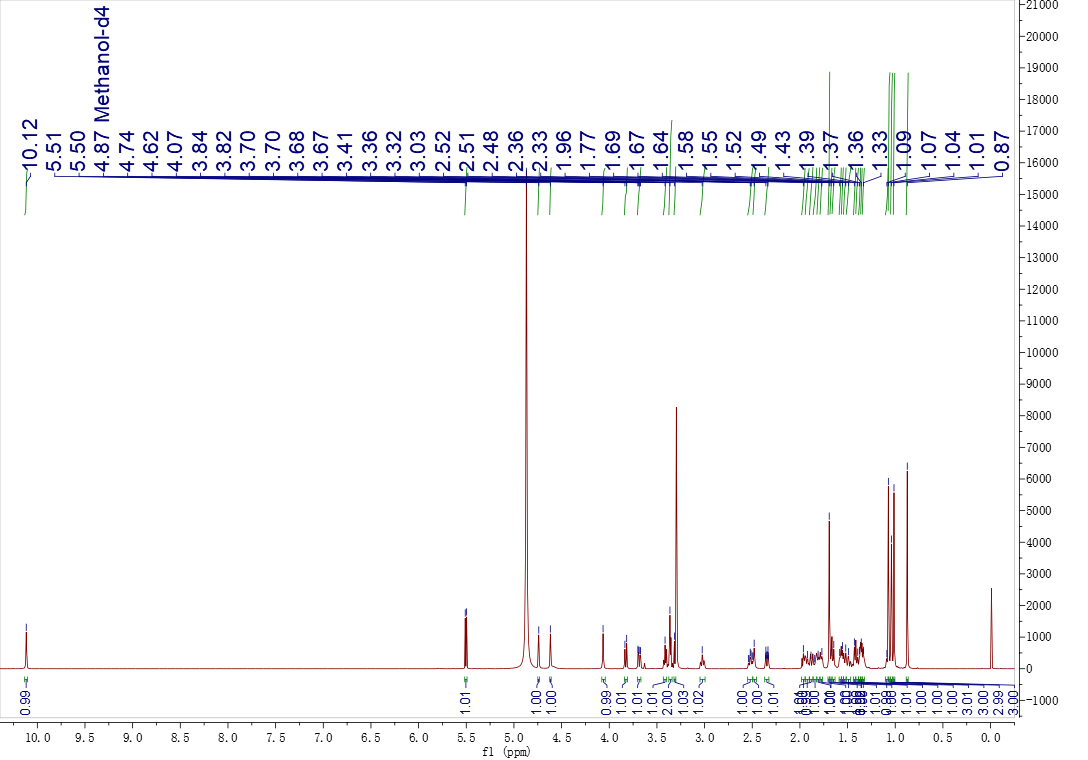


Figure. S34 ^1^H NMR spectrum of **4**


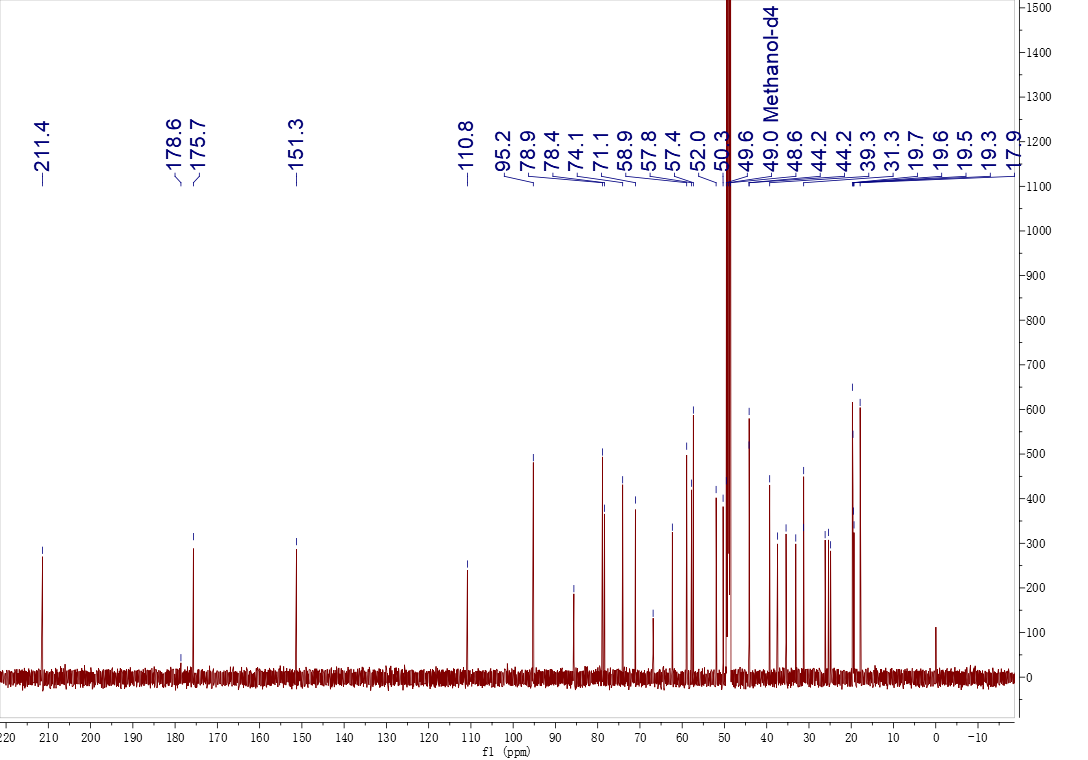


Figure. S35 ^13^C NMR spectrum of **4**


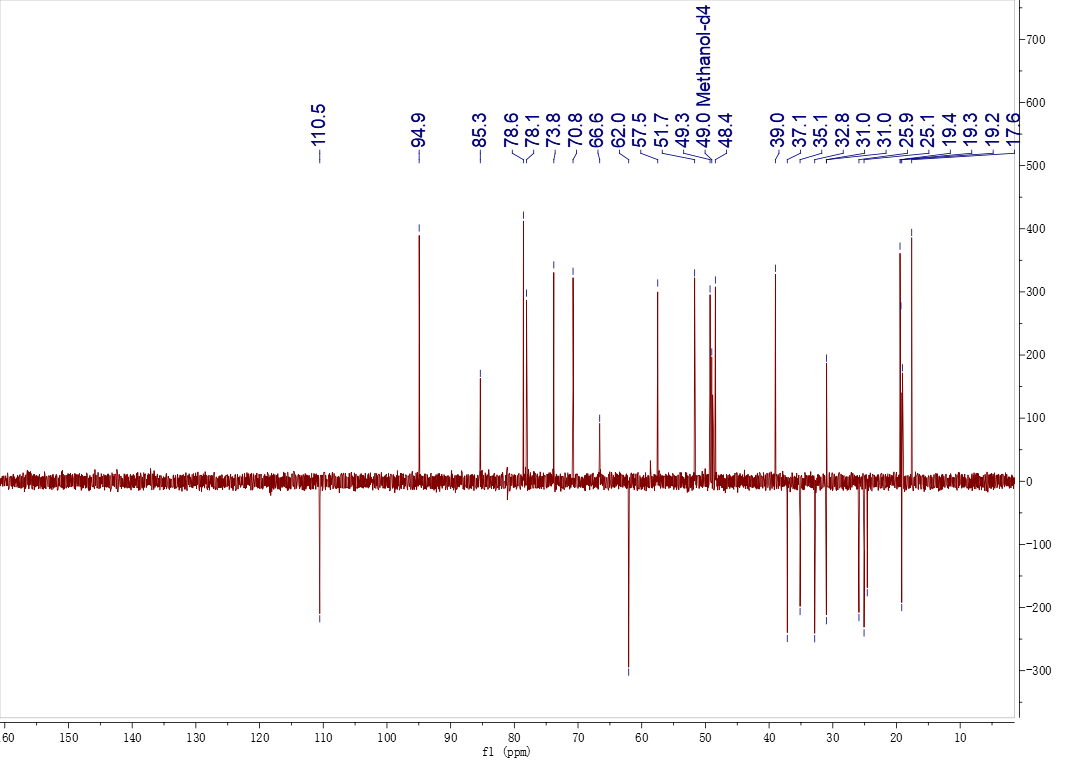


Figure. S36 DEPT-135 spectrum of **4**


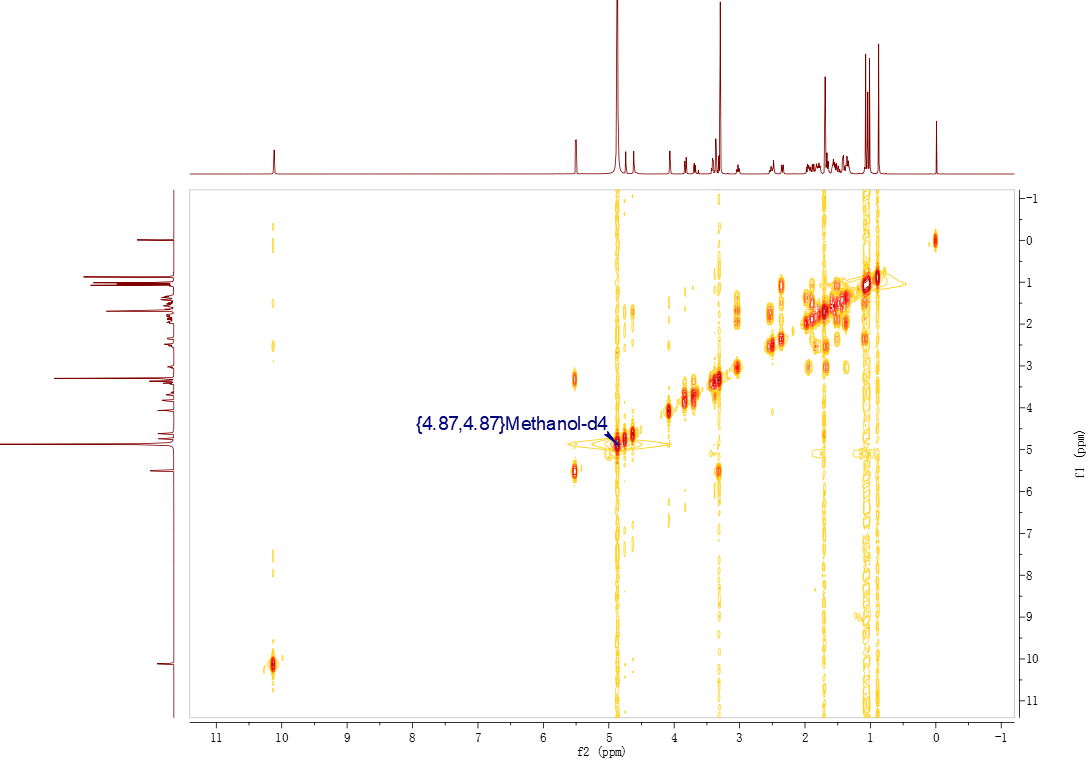


Figure. S37 ^1^H-^1^H COSY spectrum of **4**


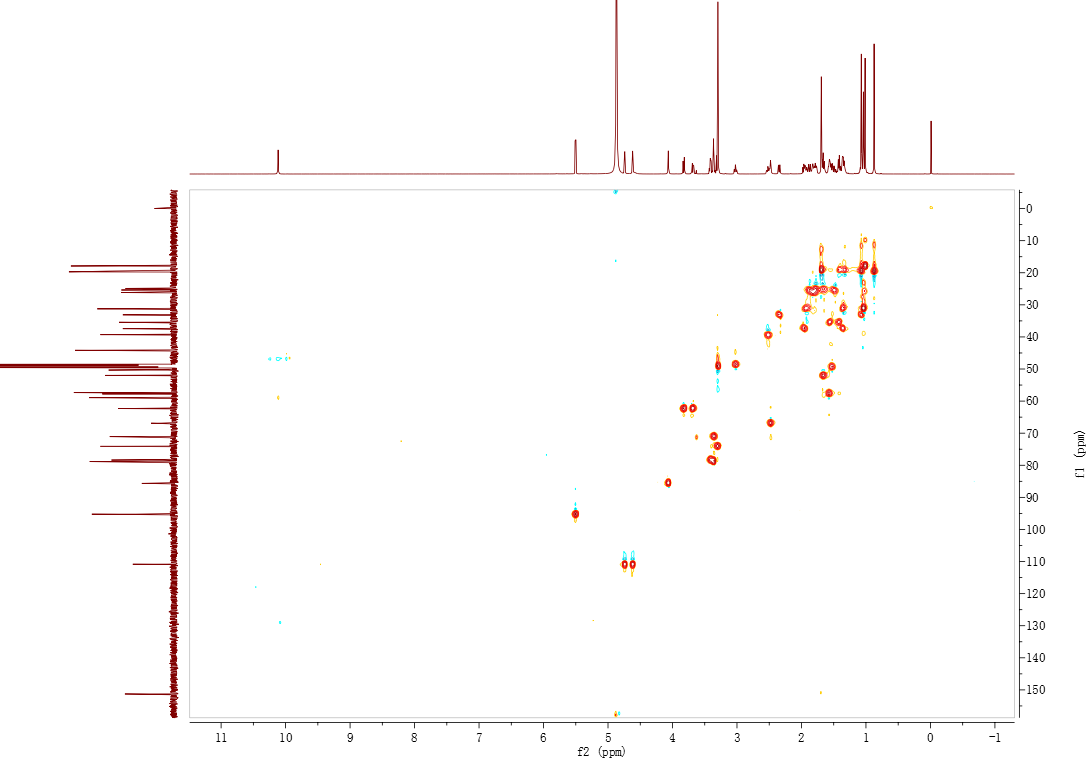


Figure. S38 HSQC spectrum of **4**


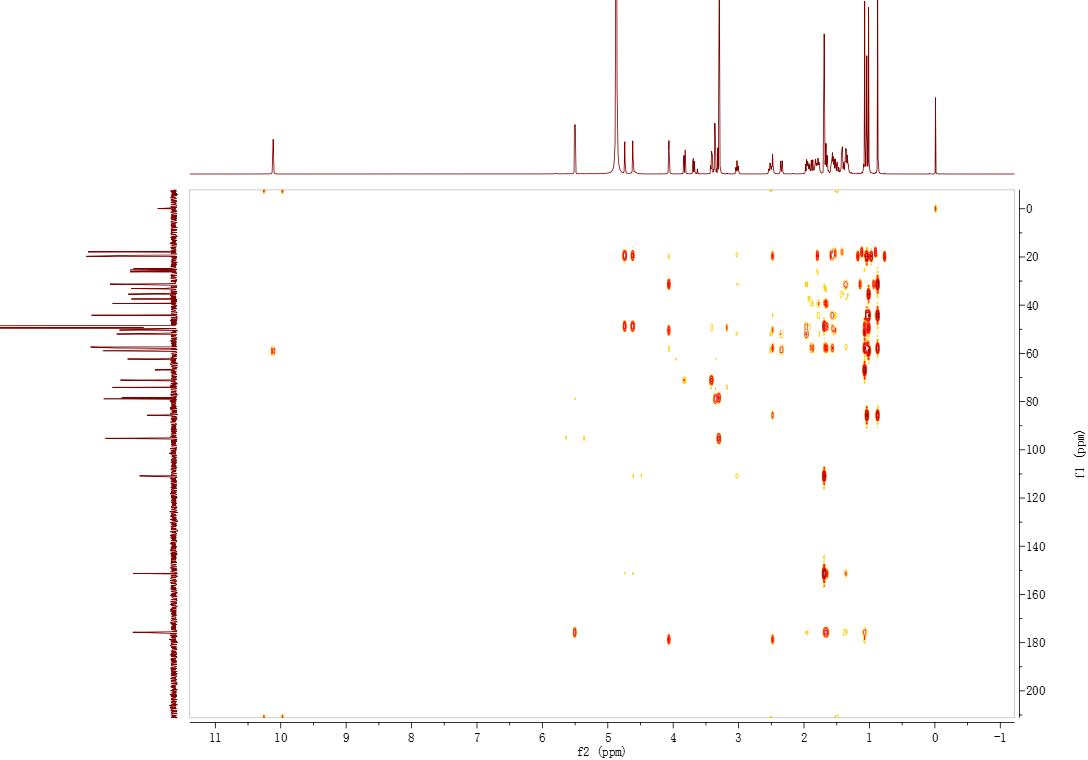


Figure. S39 HMBC spectrum of **4**


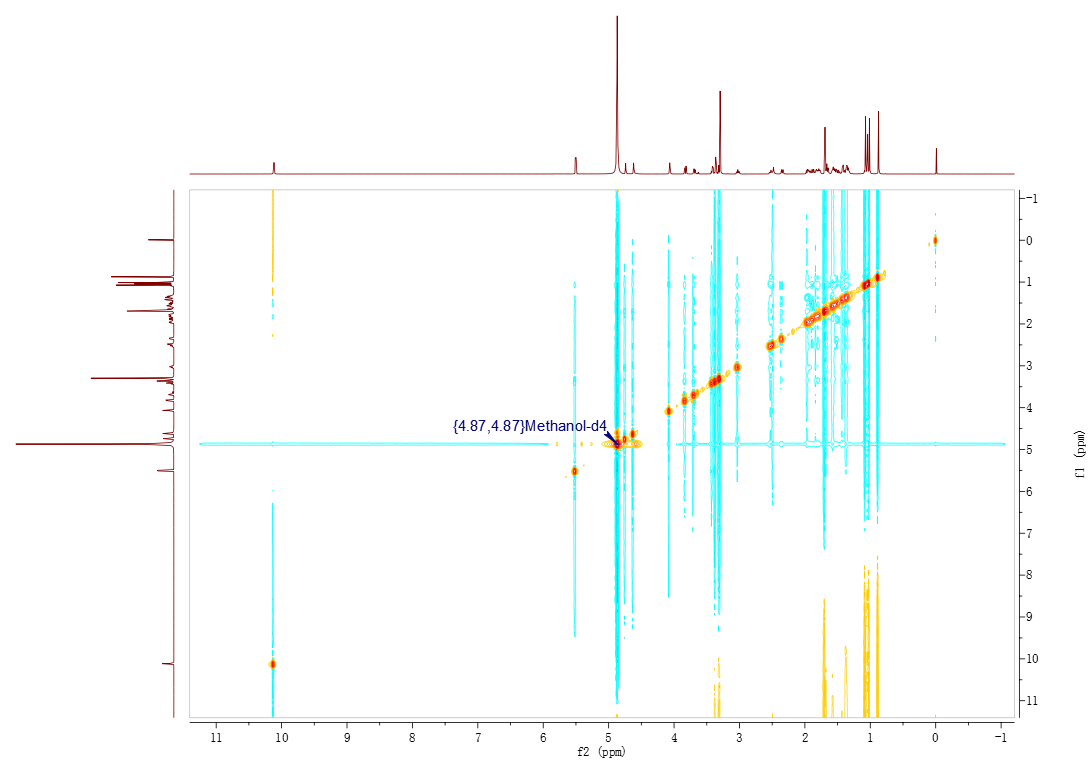


Figure. S40 NOESY spectrum of **4**
